# Useful access to enantiomerically pure protected inositols from carbohydrates: the aldohexos-5-uloses route

**DOI:** 10.3762/bjoc.12.227

**Published:** 2016-11-08

**Authors:** Felicia D’Andrea, Giorgio Catelani, Lorenzo Guazzelli, Venerando Pistarà

**Affiliations:** 1 Università di Pisa, Dipartimento di Farmacia, Via Bonanno 33, 56126 Pisa, Italy; 2 Università di Catania, Dipartimento di Scienze del Farmaco, Viale A. Doria 6, 95125 Catania, Italy

**Keywords:** aldohexos-5-uloses, inositols, intramolecular aldol condensation, stereoselective hydride reduction

## Abstract

The intramolecular aldol condensation of aldohexos-5-ulose derivatives of the D-*xylo* and L-*ribo* stereoseries has been studied. Only one of the four possible inososes was isolated from both stereoseries in reasonable yields (30–38%). The results obtained, together with the previous findings for the L-*arabino* and L-*lyxo* stereoseries, allowed for the rationalisation of a mechanism of the reaction based on open-transition-state models and electron-withdrawing inductive effects. Complementary reductions of the intermediate inososes were possible by changing the reaction conditions, and two isomeric inositol derivatives were obtained with complete stereoselection from each inosose. The presented approach permits us to control the configuration of three out of the six stereocentres of the inositol frame and gives access to seven of the nine inositols. Noteworthy, for the D-*xylo* derivative, the two-step sequence (condensation followed by reduction with NaBH(OAc)_3_) represents the biomimetic synthesis of *myo*-inositol. Furthermore, the sugar-based pathway leads directly to enantiomerically pure selectively protected inositols and does not require any desymmetrisation procedure which is needed when *myo*-inositol and other achiral precursors are employed as starting materials. As an example of application of the method, the indirect selective protection of secondary inositols’ hydroxy functions, by placing specific protecting groups on the aldohexos-5-ulose precursor has been presented.

## Introduction

**Figure 1:**
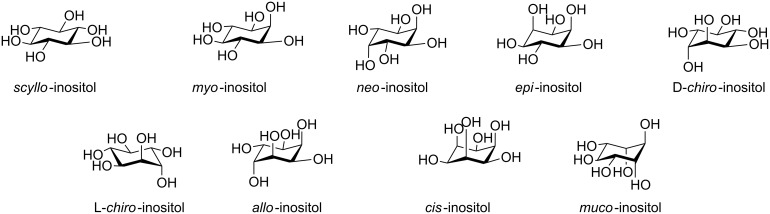
Stereoisomeric inositols.

Inositols are a family of biologically relevant compounds [1–2] constituted by nine stereoisomeric hexahydroxycyclohexanes (Figure 1): five have been found in nature (*myo*, *scyllo*, *D-chiro*, *L-chiro*, and *neo*), while the remaining four are unnatural synthetic products (*cis*, *epi*, *allo*, and *muco*).

Several *myo*-inositol phosphates, for instance D-*myo*-inositol-1,4,5-triphosphate [D-I(1,4,5)P_3_] and D-*myo*-inositol-1,3,4,5-tetrakisphosphate [D-I(1,3,4,5)P_4_], are ubiquitous in all living organisms. They are involved in different crucial cellular functions which spread from cell growth to intracellular signal transductions events [3–5]. The field investigating inositol phosphates and their involvement in mediating certain aspects of cell biology is continuously broadening. Hence, a deeper understanding of how they act on a molecular level is required.

For this reason, many research efforts were directed toward the investigation of the structure–activity relationship (SAR) between inositol phosphates and biomacromolecules. These studies require various regio- and stereoisomers of inositol phosphates [6–7] and have prompted the development of practical and efficient synthetic methods for stereoselectively accessing all natural and unnatural inositols as well as their derivatives.

There are three general synthetic approaches to prepare inositols: 1) stereoselective elaborations of the inexpensive, commercially available *myo*-inositol [8–13]; 2) elaboration of the six carbon atom skeleton of either a) tetrahydroxycyclohexene derivatives [14–15] (synthetic or natural conduritols) through stereoselective cis-hydroxylation or epoxidation–hydrolysis of the double bond or b) benzene [16–17] or halo-benzenes [18–21], by microbial oxidation; 3) carbocyclization involving organometallic intermediates (Ferrier II reaction [22] performed on 6-*O*-acyl-hex-5-enopyranosides followed by reduction [23–24]; trialkylaluminium [25–26] or titanium(IV)-assisted [27] conversion of hex-5-enopyranosides and SmI_2_-promoted [28–30] pinacol coupling of dialdehyde derivatives).

**Scheme 1:**
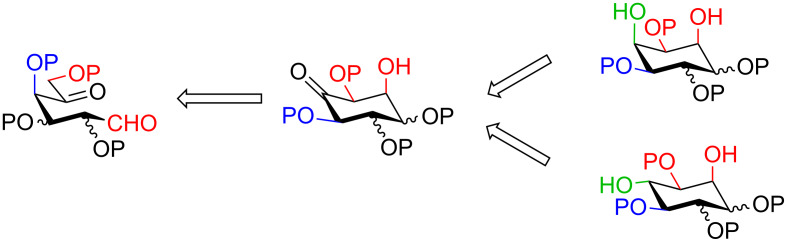
Retrosynthetic approach to inositols from aldohexos-5-uloses.

A strategy related to the latter approach relies on a base-promoted aldol condensation of aldohexos-5-uloses followed by reduction of the carbonyl group, as reported in the retrosynthetic Scheme 1. This method again uses sugars as starting material but differs from previous work in that it is metal-free.

The first application of this approach was described by Kiely [31–32] who obtained a sample of *myo*-inositol in a mixture with other non characterised stereoisomers by treating D-*xylo*-hexos-5-ulose [31] and its 6-phosphate [32] with 0.1 N aqueous NaOH followed by NaBH_4_ reduction of the crude inosose mixture.

Although these pioneering results were not of synthetic significance, they elucidated for the first time the biosynthetic correlation between D-glucose and *myo*-inositol through the intermediate formation of D-*xylo*-hexos-5-ulose. A more synthetically useful result was obtained when the aldol condensation of aldohexos-5-uloses derivatives was run in organic solvents in the presence of an organic base. The reaction occurred in a completely stereocontrolled manner and led to a single inosose intermediate [33–35]. The subsequent stereoselective reduction gave single inositols in good yields: *epi*-inositol starting from L-*arabino*-hexos-5-ulose [33] and D-*chiro*-inositol starting from the L-*lyxo* stereoisomeric dicarbonyl precursor [34].

Taking these considerations into account, we have recently completed the preparation of at least one enantioform of the four diastereoisomeric aldohexos-5-ulose series. A general approach was developed starting from β-D-galactopyranosides, including the commercially and cheaply available lactose [36–38].

Presented here is the extension of the above synthetic route to inositols using aldohexos-5-uloses derivatives of the D-*xylo* and L-*ribo* series, respectively C-4 and C-2 epimers of the L-*arabino* series. Based on these aldol condensations together with the work previously performed on the L-*arabino* [33] and L-*lyxo* [34] series, a working mechanism is presented to explain the complete stereocontrol over the formation of the two new stereogenic centres (red in Scheme 1). Furthermore, the stereochemical outcome of the intermediate inososes reduction by using different reagents, namely the stereocontrol over a third stereogenic centre (green in Scheme 1), is also reported.

## Results and Discussion

The aldol condensation reactions were performed at room temperature by treating aldohexos-5-ulose derivatives with catalytic amounts of DBU (0.15 equiv) as an organic base promoting agent in toluene, a 1:1 mixture of toluene–CH_2_Cl_2_, or CH_2_Cl_2_ to circumvent solubility problems (Table 1, see Supporting Information File 1 for full experimental data).

**Table 1:** Preparation of inosose derivatives **5–8** and **11–12** by intramolecular aldol condensation of aldohexos-5-uloses derivatives **1–4** and **9–10**.

Compound	Conditions^a^	Products (% yield)

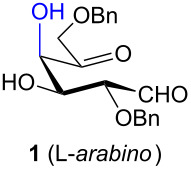	5% DBU solution, dry toluene, rt, 6 h	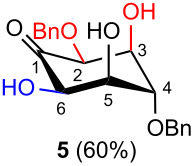
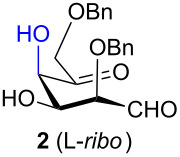	5% DBU solution, dry CH_2_Cl_2_, 0 °C, 1.5 h	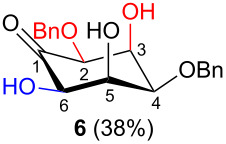
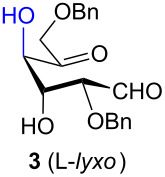	5% DBU solution, dry 1:1 toluene/CH_2_Cl_2_, rt, 2.5 h	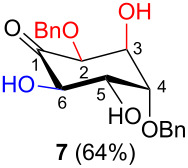
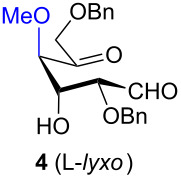	5% DBU solution, dry CH_2_Cl_2_, rt, 2.0 h	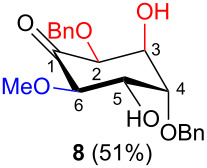
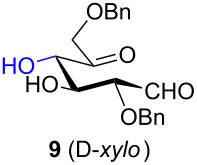	5% DBU solution, dry CH_2_Cl_2_, rt, 1 h	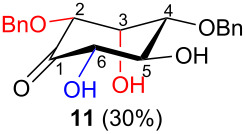
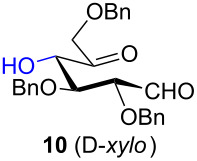	5% DBU solution, dry 1:1 toluene/CH_2_Cl_2_, rt, 3 h	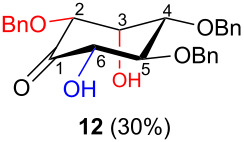

^a^Intramolecular cyclizations of **1** [33], **3** [34], and **4** [35] have been reported in previous works.

In all the cases studied, only one of the four possible inososes was isolated. This was characterised by a *cis* axial–equatorial arrangement of the two oxygenated substituents on the new stereocentres (2 and 3) which were also *cis* to the other substituent α to the keto group (position 6). This 2,3,6-*cis* arrangement of the aldol products was observed irrespective to the stereochemistry of the other two positions (4 and 5), corresponding to the C-2 and C-3 of the parent dicarbonyl derivative.

To determine the effect of additional protecting groups on the reaction, the intramolecular aldol condensation was also performed on aldohexos-5-uloses of the L-*lyxo* and D-*xylo* series bearing a methyl protection on position 4 or a benzyl protection on position 3 (compounds **4** [35] and **10** [38], respectively). The stereo-outcome and the yields confirmed the cyclisation results obtained for dibenzyl derivatives.

Considering the results obtained, the intramolecular aldol condensation of the *lyxo* and *arabino* stereoseries affords the inososes in fairly good yields (54–60%) [33–35], while the same reaction run on the *ribo* and *xylo* stereoseries gives the cyclization products only in moderate yields (30–38%). In these latter cases, the colour of the reaction mixture changed over time from light yellow to dark orange. NMR analysis of the crude mixture verified the formation of a single inosose, which was the one isolated after chromatographic purification, together with indistinct side products possibly deriving by polycondensations or eliminations after the aldol condensation.

The structure and stereochemistry of inososes **5–8**, **11**, and **12** were confirmed by ^1^H, ^13^C and 2D NMR experiments. For example, the conformation of inosose **6** was established by the similar value of the vicinal proton coupling constants and by the presence of long-range coupling between H-2 and H-6 (*J*_2,6_ = 1.4 Hz), and H-3 and H-5 (*J*_3,5_ = 2.6 Hz). For **11** and **12**, the high value of the *J*_5,6_ (9.4 Hz and 8.5 Hz, respectively) and *J*_4,5_ (9.7 Hz and 9.3 Hz), and the low value of the *J*_2,3_ (2.4 Hz and 2.7 Hz) and *J*_3,4_ (2.2 Hz and 2.3 Hz) agree with four substituents in the equatorial position for the preferred conformer.

A comparable example of intramolecular aldol condensation of a 1,5-dicarbonyl derivative has been reported by Tadano et al. [39]. Almost a sole aldol product was obtained in a good yield and was characterised by the same 2,3,6-*cis* relationship observed in our experiments. In this case, the authors focused their attention only on the new formed chiral centres and on the *cis*-orientation of their substituents. To rationalise the result obtained, they referred to the topological rules, which were proposed by Seebach and Golinski [40] to explain the preferred formation of the *threo*-configuration in intermolecular Michael additions. Unfortunately, not all these rules are applicable to the intramolecular reaction under investigation and, even more disappointing, they do not give any correlation between the two new chiral centres and the chiral centre α to the keto group which was already present on the parent dicarbonyl compound. Indeed, this appears as the most relevant and unexpected result of the present work. We performed the intramolecular aldol condensation on 4- and 6-deoxy-aldohexos-5-uloses of the L-*arabino* series (not shown). In the latter case, no reaction was observed even after longer reaction times. On the contrary, the C-4 deoxygenated compound afforded an inseparable complex mixture of products, possibly derived from a first carbocyclisation followed by elimination reactions.

In an attempt to find an explanation of the stereochemical outcome of the reaction, we directed our attention toward the open-transition-state models. These have been proposed to explain the prevalent formation of *syn* products, irrespective of the enolate geometry [41], in aldol reactions performed in the absence of a coordinating metal center (for instance, in the case of tin and zirconium enolates, and of “naked” enolates generated from enolsilanes [42]).

In the open-transition-state model, the enolate and the carbonyl group are orientated in an antiperiplanar fashion, maximazing the distance between the negatively charged oxygen atoms.

**Figure 2:**
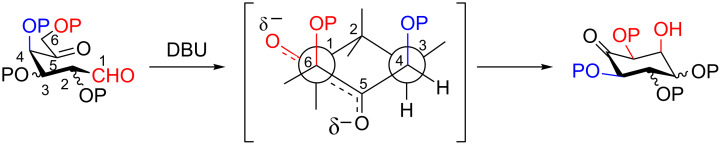
Hypothesis of the preferred transition state.

On these bases, a possible transition state for the intramolecular reaction, which considers also the 1,4,6-*cis* relationship (numbering referred to the dicarbonyl compound), is reported in Figure 2.

A working hypothesis is that the axial arrangement of electron-withdrawing substituents in positions 4 and 6 diminishes the negative charge density on the 5-oxygen atom and decreases in this way the overall energy of the transition state, thereby favouring the intramolecular reaction. These kind of inductive effects in cyclic system has been reported before [43–44].

The structure of every inosose obtained allows for complementary stereoselective reductions by choosing the proper reaction conditions. In fact (Table 1), the substituents α to the keto function (2 and 6) and the axial OH in position 3 have always a *cis* orientation which would favour an equatorially directed *anti* reduction using NaBH_4_ as a reducing reagent in an alcoholic solvent (condition **A**). In particular, it has been proven that the axial OH group β to the keto function plays a fundamental role in directing the nucleophilic attack [45–47]. It has also to be mentioned that by changing the experimental conditions (temperature and alcoholic solvent) a different diastereoselectivity has been reported in the reduction of the same protected inosose [45,48]. This highlights how subtle changes in the inosose structure and/or in the experimental conditions affect the stereo-outcome of the reduction.

**Figure 3:**
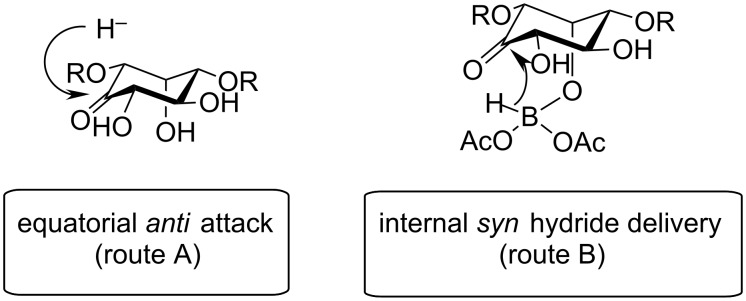
Stereoselective reduction of inosose intermediate.

Also, the same free axial hydroxy function, deriving from the aldehyde which is beta to the carbonyl group (position 3), would allow for the complementary axial directed *syn* reduction using NaBH(OAc)_3_ in an AcOH–CH_3_CN mixture through an intramolecular hydride transfer as reported by Evans [49] (condition B). The axial OH function would be able to coordinate the reducing agent and thus give an internal hydride transfer from the same side through a six membered cyclic transition state (Figure 3).

In Table 2, the results of the complementary reductions (conditions A and B) are summarised (see Supporting Information File 1 for full experimental data). Seven different inositol derivatives **13–19** were obtained from the four inososes **5–7** and **11** in a stereoselective way, thereby controlling the stereochemistry of a third stereocentre of the inositol frame. The results of the reduction reactions are in good agreement with what has been reported before on similar compounds [45]. The structure and stereochemistry of inositols **13–19** were confirmed by ^1^H, ^13^C and 2D NMR experiments directly or after per-acetylation of the reduction product and/or through comparison with previously reported compounds.

**Table 2:** Stereoselective complementary reductions (conditions A and B) of inososes **5–7** and **11**^a,b^.

Compound	Reduction conditions	Inositol derivatives	Configuration

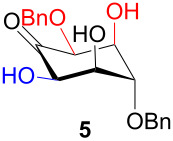	A: NaBH_4_, EtOH, −78 °C to 15 °C	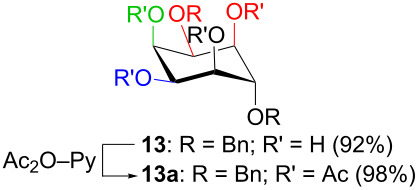	*epi*
	B: NaBH(OAc)_3_, AcOH, CH_3_CN, rt	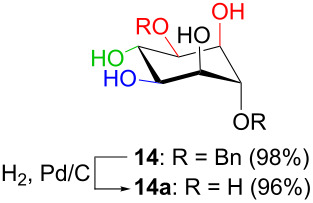	*muco*
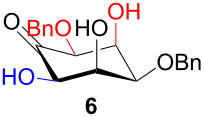	A: 1) NaBH_4_, MeOH, 0 °C; 2) Ac_2_O, pyridine	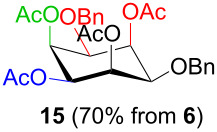	*cis*
	B: NaBH(OAc)_3_, AcOH, CH_3_CN, rt	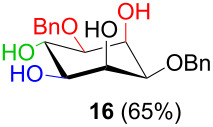	*epi*
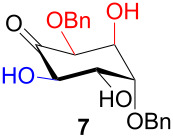	A: NaBH_4_, EtOH, −78 °C to 15 °C	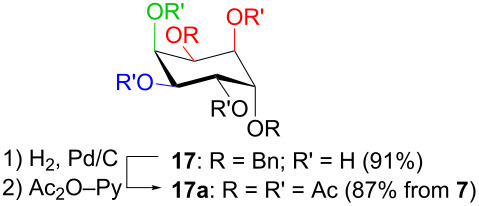	*allo*
	B: NaBH(OAc)_3_, AcOH, CH_3_CN, rt	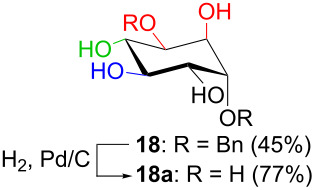	*D-chiro*
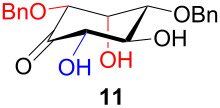	A: NaBH_4_, MeOH, 0 °C	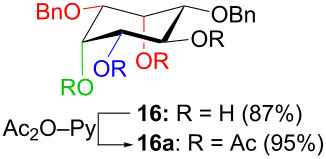	*epi*
	B: NaBH(OAc)_3_, AcOH, CH_3_CN, rt	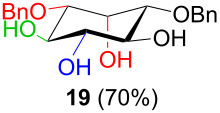	*myo*

^a^Inositol derivatives in the table are represented in the way they are obtained as a result of the stereoselective reduction and the preferred conformations can be deduced from the experimental section. ^b^Preparation of **13** [33], **13a** [33], **17a** [34] and **18a** [34] has been described in previous works.

Noteworthy, the two step sequence performed on the D-*xylo* derivative **11** leads to *myo*-inositol derivative **19** when NaBH(OAc)_3_ is employed in the stereoselective reduction of the inosose and represents the biomimetic conversion of D-*xylo*-aldohexos-5-ulose derivatives into *myo*-inositol derivatives.

**Scheme 2:**
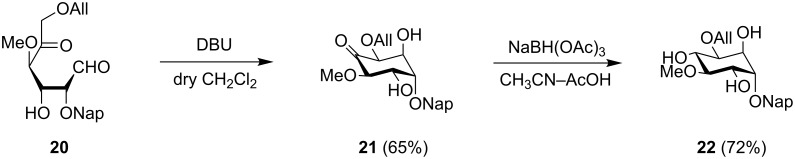
Intramolecular cyclization of an orthogonally protected L-*lyxo*-aldohexos-5-ulose derivative.

As well as the stereoselective synthesis of enantiopure inositol derivatives, also the possibility to pick and tag specific positions of the inositol ring, installing the protective groups earlier on the carbohydrate frame through the well-known sugar chemistry, is of extreme interest. Although some attractive findings have been reported so far [46–47], the regioselective protection of inositols is still a troublesome area due to the comparable reactivity of the secondary hydroxy functions. Therefore, the carbocyclization of a selectively protected dicarbonyl sugar, namely the L-*lyxo* derivative **20**, whose synthesis will be presented in a forthcoming paper, has been investigated (Scheme 2).

The expected inosose **21** was obtained under standard conditions and was then submitted to the reduction step with NaBH(OAc)_3_. The D-*chiro* derivative **22** was isolated in a 47% yield over two steps and with complete stereoselectivity in accordance with the predicted stereo-outcome.

In this way, the orthogonal protecting groups (allyl and naphthalenylmethyl) installed on aldohexos-5-ulose **20**, differentiated the two 1,2-*cis* hydroxy couples (2,3 and 4,5 positions) of **22** which can be easily transformed further in a selective manner.

## Conclusion

The intramolecular aldol condensation of aldohexos-5-ulose derivatives of the four stereoseries has been studied. By obtaining the inosose derivatives shown, a better understanding of the relative influence of each position on the stereochemical course of the reaction was gained. These findings allowed us to formulate a plausible rationalization of the mechanism based on open-transition-state models and on inductive effects of axial *anti* arranged electron-withdrawing substituents.

The overall process, condensation followed by reduction, which involves the formation of three new stereocentres, represents a general access to inositol derivatives. In particular, in a complete stereoselective fashion, inositols of six different configurations characterised by the 2,3,6-*cis* arrangement of three substituents were obtained from aldohexos-5-uloses. Furthermore, the formal synthesis of L-*chiro*-inositol could be considered as achieved starting from known D-*lyxo*-aldohexos-5-ulose derivatives [37,50]. Even if in some cases yields of isolated compounds are not too high, this sugar-based approach gives access directly to enantiomerically pure inositol derivatives. This avoids time and cost-consuming desymmetrisation procedures required when *myo*-inositol is used as starting material in the preparation of inositol derivatives.

In addition, introducing a suitable pattern of protecting groups in the sugar frame, which will then be present in the inositol ring, has been shown. This allows for an indirect regioselective differentiation of the inositols’ secondary hydroxy groups, which is difficult to achieve by common chemical means.

## Supporting Information

File 1 Experimental procedures, characterization data of new compounds and ^1^H and ^13^C NMR spectra of compounds **6**, **11**, **12**, **14–16**, **16a**, **18**, **19** and **21**, **22**.
