# Supplementary material for: Useful access to enantiomerically pure protected inositols from carbohydrates: the aldohexos-5-uloses route
Source: Beilstein J Org Chem. 2016 Nov 8;12:2343–50. doi: 10.3762/bjoc.12.227 (PMC5238563; doi:10.3762/bjoc.12.227)
Supplement: File 1 — Experimental procedures, characterization data of new compounds and 1H and 13C NMR spectra of compounds 6, 11, 12, 14–16, 16a, 18, 19 and 21, 22. [file Beilstein_J_Org_Chem-12-2343-s001.pdf]

Supporting Information File 1

for

Useful Access to Enantiomerically Pure Protected Inositols from Carbohydrates: the Aldohexos-5-uloses Route

Felicia D'Andrea,[1] Giorgio Catelani,[1] Lorenzo Guazzelli,*,[1] Venerando Pistarà[2]

[1]*Università di Pisa, Dipartimento di Farmacia, Via Bonanno 33, 56126 Pisa, Italy*

[2]*Università di Catania, Dipartimento di Scienze del Farmaco, Viale A. Doria 6, 95125 Catania, Italy*

**Corresponding author*

Email: lorenzo.guazzelli@unipi.it;

Experimental procedures, characterization data of new compounds and ^{1}H and ^{13}C NMR spectra of compounds 6, 11-12, 14-16, 16a, 18-19 and 21-22.

Contents

General Methods	S2
Experimental procedures and physico-chemical characterization	S2-S13
References	S13
^{1}H- and ^{13}C-NMR spectra of **6**	S14
^{1}H- and ^{13}C-NMR spectra of **11**	S15
^{1}H- and ^{13}C-NMR spectra of **12**	S16
^{1}H- and APT-NMR spectra of **14**	S17
^{1}H- and ^{13}C-NMR spectra of **15**	S18
^{1}H- and ^{13}C-NMR spectra of **16**	S19
^{1}H- and ^{13}C-NMR spectra of **16a**	S20
^{1}H-, ^{13}C-NMR and DEPT-135 spectra of **18**	S21-S22
^{1}H- and ^{13}C-NMR spectra of **19**	S22-S23
^{1}H- and ^{13}C-NMR spectra of **21**	S23-S24
^{1}H- and ^{13}C-NMR spectra of **22**	S24-S25

General Methods

Melting points were determined with a Kofler hot-stage apparatus and are uncorrected. Optical rotations were measured on a Perkin-Elmer 241 polarimeter at 20±2 °C. ^{1}H NMR spectra were recorded in appropriate solvents with a Bruker AC 200 instrument and a Bruker Avance II operating at 200 MHz and 250.13 MHz respectively and with a Varian VnmrJ instrument operating at 500 MHz. ^{13}C NMR spectra were recorded with the spectrometers operating at 50 or 62.9 or 125 MHz. The assignments were made, when possible, with the aid of DEPT, HETCOR, COSY experiments. The first order proton chemical shifts δ are referenced to either residual CD_3CN (δ_H 1.94, δ_C 1.28) or residual CD_3OD (δ_H 3.31, δ_C 49.0) and *J*-values are given in Hz. All reactions were followed by TLC on Kieselgel 60 F_{254} with detection by UV light and/or with ethanolic 10% phosphomolybdic or sulfuric acid, and heating. Kieselgel 60 (E. Merck, 70-230 and 230-400 mesh, respectively) was used for column and flash chromatography. Solvents were dried by distillation according to standard procedures, and storage over 4Å molecular sieves activated for at least 24 h at 200 °C. All reagents were purchased from Aldrich Chemical Co. and were used without further purification. $MgSO_4$ was used as the drying agent for solutions. Compounds 2,6-di-*O*-benzyl-L-*ribo*-aldohexose-5-ulose (**2**) [1], 2,4-di-*O*-benzyl-2L-(2,3,5,6/4)-pentahydroxy-cyclohexanone (**5**) [2], 2,6-di-*O*-benzyl-D-*xylo*-aldohexose-5-ulose (**9**) [3], 2,3,6-tri-*O*-benzyl-D-*xylo*-aldohexose-5-ulose (**10**) [1], were prepared according to the reported procedures.

Experimental procedures and physico-chemical characterization

General Procedure for aldol condensation of 2, 9 and 10.

To a solution of the appropriate 1,5-dicarbonyl-hexose (1.0 mmol) in either dry CH_2Cl_2 (34 mL, for **2** and **9**) or a 1:1 mixture of dry toluene-CH_2Cl_2 (28 mL, for **10**), under Argon atmosphere, a 5% DBU solution in dry CH_2Cl_2 (0.72 mL) was added and the mixture was stirred either at room temperature (for **9** and **10**) or 0 °C (for **2**) until the starting material was disappeared (1-6 h, TLC). A 0.5% AcOH solution in dry CH_2Cl_2 (0.5 mL) was added and after 10 min the mixture was concentrated under diminished pressure and repeatedly co-evaporated with toluene (4 × 30 mL). Purification of crude product by flash chromatography on silica gel afforded pure inososes **6**, **11** and **12**.

2,4-di-O-benzyl-2L-(2,3,4,5/0)-pentahydroxycyclohexanone (6).

Aldol condensation of **2** (116 mg, 0.32 mmol) was performed in dry CH_2Cl_2 (11 mL) in accordance with the general procedure and the reaction was stopped after 1.5 h. Purification of the crude product by flash chromatography on silica gel (3:7 hexane-EtOAc) gave pure **6** (44 mg, 38%) as a clear syrup: R_f 0.22 (2:8 hexane-EtOAc); $[\alpha]_D^{23}$ -18.8 (c 0.96, $CHCl_3$); ^{1}H NMR (250.13 MHz, CD_3CN-D_2O) δ: 7.34-7.20 (m, 10H, Ar-*H*), 4.77, 4.63 (AB system, 2H, $J_{A,B}$=11.6 Hz, C*H*$_2$Ph), 4.68, 4.47 (AB system, 2H, $J_{A,B}$=11.9 Hz, C*H*$_2$Ph), 4.55 (dt, 1H, $J_{3,4}$=$J_{3,5}$=2.6 Hz, $J_{2,3}$=3.4 Hz, H-3), 4.41 (dt, 1H, $J_{4,5}$=$J_{3,5}$=2.6 Hz, $J_{5,6}$=3.7 Hz, H-5), 4.26 (dd, 1H, $J_{2,6}$=1.4 Hz, H-6), 4.21 (dd, 1H, H-2), 3.87 (t, 1H, H-4); ^{13}C NMR (62.9 MHz, CD_3CN-D_2O) δ: 207.5 (C-1), 139.0, 138.8 (Ar-*C*), 129.4-128.8 (Ar-*C*H), 81.6 (C-2), 76.7 (C-5), 75.5 (C-3), 75.4 (C-6), 73.8 (C-4), 72.5, 71.3 (2 × *C*H$_2$Ph). Anal. Calcd for $C_{20}H_{22}O_6$: C, 67.03; H, 6.19. Found: C, 66.98; H, 6.20.

2,4-di-O-benzyl-2D-(2,3,4,6/5)-pentahydroxycyclohexanone (11).

Aldol condensation of **9** (99.3 mg, 0.28 mmol) was performed in dry CH_2Cl_2 (9.5 mL) in accordance with the general procedure and the reaction was stopped after 1 h.

Purification of the crude product by flash chromatography on silica gel (95:5 $CHCl_3$-MeOH) gave pure **11** (29.4 mg, 30% yield) as a syrup: R_f 0.18 (2:8 hexane-EtOAc); $[\alpha]_D^{23}$ +12.2 (c 0.96, $CHCl_3$); ^{1}H NMR (250.13 MHz, D_2O) δ: 7.34-7.30 (m, 10H, Ar-*H*), 4.65-4.55 (m, 4H, 2 × C*H*$_2$Ph), 4.23 (dd, 1H, $J_{2,3}$=2.4 Hz, $J_{3,4}$=2.2 Hz, H-3), 4.15 (bd, 1H, H-2), 4.02 (d, 1H, $J_{5,6}$=9.4 Hz, H-6), 3.72 (dd, 1H, $J_{4,5}$=9.7 Hz, H-5), 3.59 (dd, 1H, H-4); ^{13}C NMR (62.9 MHz, D_2O) δ: 206.1 (C-1), 138.3, 137.8 (Ar-*C*), 129.5-129.1 (Ar-*C*H), 80.7 (C-2), 78.7 (C-4), 77.0 (C-6), 74.9 (C-5) 72.8, 72.3 (2 x *C*H$_2$Ph), 69.4 (C-3). Anal. Calcd for $C_{20}H_{22}O_6$: C, 67.03; H, 6.19. Found: C, 66.95; H, 6.21.

2,4,5-tri-O-benzyl-2D-(2,3,4,6/5)-pentahydroxycyclohexanone (12).

Aldol condensation of **10** (127.6 mg, 0.28 mmol) was performed in a dry 1:1 mixture of toluene-CH_2Cl_2 (8 mL) in accordance with the general procedure and the reaction was stopped after 3 h. Purification of the crude product by flash chromatography on silica gel (9:1 $CHCl_3$-MeOH) gave pure **12** (38.3 mg, 30% yield) as a syrup: R_f 0.42 (1:1 toluene-CH_2Cl_2); $[\alpha]_D^{23}$ +14.8 (c 1.11, $CHCl_3$); ^{1}H NMR (250.13 MHz, CD_3CN) δ: 7.44-7.22 (m, 15H, Ar-*H*), 4.83, 4.79 (AB system, 2H, $J_{A,B}$=11.2 Hz, C*H*$_2$Ph), 4.76, 4.66 (AB system, 2H, $J_{A,B}$=11.7 Hz, C*H*$_2$Ph), 4.73, 4.51 (AB system, 2H, $J_{A,B}$=11.7 Hz, C*H*$_2$Ph), 4.45 (dd, 1H, $J_{3,4}$=2.3 Hz, $J_{2,3}$=2.7 Hz, H-3), 4.28 (dd, 1H, $J_{2,6}$=1.5 Hz, H-2), 4.18 (dd, 1H, $J_{5,6}$=8.5 Hz, H-6), 3.83 (dd, 1H, $J_{4,5}$=9.3 Hz, H-4), 3.74 (dd, 1H, H-5); ^{13}C NMR (62.9 MHz, CD_3CN) δ: 205.6 (C-1), 140.2, 139.6, 139.2 (3 x Ar-*C*), 129.2-127.6 (Ar-*C*H), 84.6 (C-5), 81.7 (C-2), 79.9 (C-4), 77.9 (C-6) 75.6, 72.8, 72.6 (3 × *C*H$_2$Ph), 70.5 (C-3). Anal. Calcd for $C_{27}H_{28}O_6$: C, 72.30; H, 6.29. Found: C, 72.28; H, 6.27.

2-O-allyl-4-O-naphthalenylmethyl-6-O-methyl-2L-(2,3,6/4,5)-pentahydroxycyclohexa-none (21).

Aldol condensation of **20** (365 mg, 0.98 mmol) was performed in dry CH_2Cl_2 (24 mL) with DBU (5% solution in dry CH_2Cl_2, 0.38 mL) in accordance with the general procedure. The reaction was stirred until the starting material was disappeared (2.5 h, TLC, 4:6 hexane-EtOAc). Purification of the crude product by flash chromatography on silica gel (4:6 hexane-EtOAc) gave pure inosose **21** (237 mg, 65% yield) as a white foam: R_f 0.19 (4:6 hexane-EtOAc); $[\alpha]_D^{23}$ +30.6 (*c* 0.98, $CHCl_3$); ^1H NMR (CD_3CN, 250.13 MHz,) δ: 7.90 (m, 4H, Ar-*H*), 7.52 (m, 3H, Ar-*H*), 5.98 (ddt, 1H, J_{trans}=17.1 Hz, J_{cis}=10.4 Hz, *J*=5.2 Hz, C*H*=), 5.27 (dq, 1H, J_{trans}=17.3 Hz, *J*=1.7 Hz, CH_2=), 5.13 (dq, 1H, J_{cis}=10.4 Hz, *J*=1.4 Hz, CH_2=), 4.99, 4.87 (AB system, 2H, J_{AB}=12.0 Hz, CH_2Nap), 4.36 (dd, 1H, $J_{2,3}$=3.3 Hz, $J_{2,6}$=1.5 Hz, H-2), 4.28 (bt, 1H, $J_{3,4}$=$J_{3,OH}$=3.5 Hz, H-3), 4.18 (ddt, 1H, *J*=1.5 Hz, *J*=5.2 Hz, *J*=13.1 Hz, CH_2O), 4.03 (dd, 1H, $J_{5,6}$=9.9 Hz, H-6), 3.96 (ddt, 1H, *J*=1.4 Hz, *J*=5.7 Hz, *J*=13.1 Hz, CH_2O), 3.96 (dd, 1H, $J_{4,5}$=3.2 Hz, H-4), 3.84 (ddd, 1H, $J_{5,OH}$=6.2 Hz, H-5), 3.50 (d, 1H, OH-5), 3.49 (d, 1H, OH-3), 3.43 (s, 3H, OCH_3); ^{13}C NMR (CD_3CN, 62.9 MHz,) δ: 204.6 (C-1), 137.2, 134.2, 133.9 (3 $\times$ Ar-*C*), 135.8 (*C*H=), 128.9-126.9 (Ar-*C*H), 117.2 (*C*H_2=), 86.2 (C-6), 81.9 (C-2), 79.5 (C-4), 74.6 (*C*H_2Nap), 73.5 (C-5), 71.5 (*C*H_2O), 71.3 (C-3), 59.4 (O*C*H_3). Anal. Calcd for $C_{21}H_{24}O_6$: C, 67.63; H, 6.50. Found: C, 67.67; H, 6.54.

1,3-di-*O*-Benzyl-1L-*muco*-inositol (14).

To a solution of **5** (100 mg, 0.28 mmol) in CH_3CN (5 mL), AcOH (0.4 mL) and $NaBH(OAc)_3$ (106 mg, 0.5 mmol) were consecutively added. The mixture was stirred at room temperature until the starting material was disappeared (50 min, TLC, 2:8 hexane-EtOAc). Excess of hydride was decomposed with a solution of aq $NaHSO_4$ (0.5 M) and the reaction mixture was repeatedly coevapored with toluene (3 $\times$ 20 mL) under diminished pressure. The residue was partitioned between brine (20 mL)

and EtOAc (20 mL), the organic phase was separated and the aq layer extracted with EtOAc (3 × 30 mL). The combined organic phases were collected, dried, filtered and concentrated under diminished pressure and afforded a residue (93 mg, 98% yield) constituted (NMR) exclusively by **14** as a clear syrup: R_f 0.31 (EtOAc); $[\alpha]_D^{23}$ -32.2 (*c* 0.47, $CHCl_3$); ^{1}H NMR (500 MHz, CD_3CN-D_2O) δ: 7.43-7.29 (m, 10H, Ar-*H*), 4.68, 4.62 (AB system, 2H, $J_{A,B}$=12.0 Hz, C*H2*Ph), 4.61, 4.53 (AB system, 2H, $J_{A,B}$=11.5 Hz, C*H2*Ph), 4.10 (bt, 1H, $J_{3,4}$=$J_{4,5}$=3.5 Hz, H-4), 3.93 (bt, 1H, $J_{3.4}$=$J_{2,3}$=4.0 Hz, H-3), 3.88 (t, 1H, $J_{1,6}$=$J_{5,6}$=9.0 Hz, H-6), 3.79 (t, 1H, $J_{1,2}$=$J_{2,3}$=4.0 Hz, H-2), 3.55 (dd, 1H, H-5), 3.48 (dd, 1H, H-1);^{13}C NMR (125 MHz, CD_3CN-D_2O) δ: 139.2, 138.9 (2 × Ar-*C*), 128.8-128.4 (Ar-*C*H), 80.4 (C-1), 77.8 (C-2), 73.2 (C-5), 72.7, 72.3 (2 × *C*H_2Ph), 72.4 (C-3), 70.3 (C-6), 69.9 (C-4). Anal. Calcd for $C_{20}H_{24}O_6$: C, 66.65; H, 6.71. Found: C, 66.73; H, 6.66.

Muco-*inositol (14a).*

To a solution of **14** (60 mg, 0.167 mmol) in dry MeOH (5 mL), 10% Pd on charcoal (20 mg) was added and the mixture was stirred at room temperature under H_2 (atmospheric pressure) until the starting compound was completely reacted (TLC, 12 h). The suspension was diluted with MeOH (15 mL), filtered over a pad of Celite®, washed with MeOH, and the combined organic phases were concentrated at diminished pressure. The solid residue (28.8 mg, 96% yield) was constituted (NMR) exclusively by **14a**. Crystallization (EtOH) afforded **14a** pure as a white solid: mp 287-295 °C (EtOH); Lit [4] mp 285-300 °C; ^{1}H NMR (200 MHz, D_2O) δ: 3.84 (bt, 2H, spl. ≅ 6.0 Hz, H-3, H-6); 3.73 (bd, 4H, spl. ≅ 5.9 Hz, H-1, H-2, H-4, H-5); ^{13}C NMR (50 MHz, D_2O) δ: 72.8 (C-1, C-2, C-4, C-5), 70.8 (C-3, C-6). Anal. Calcd for $C_6H_{12}O_6$:

C, 40.00; H, 6.71. Found: C, 40.17; H, 6.85. ^{1}H and ^{13}C NMR data were in good agreement with those reported [5-6].

1,2,3,5-tetra-*O*-Acetyl-4,6-di-*O*-benzyl-*cis*-inositol (15).

A solution of **6** (78 mg, 0.22 mmol) in MeOH (5 mL) was treated, at 0 °C, with $NaBH_4$ (35.2 mg, 4 equiv). The mixture was stirred at 0 °C until the starting material was disappeared (30 min, TLC, 9:1 CH_2Cl_2-MeOH). Amberlist 15 was added to neutralise; the mixture was filtered and the solvent was removed under diminished pressure. The crude residue was dissolved in a 1:2 mixture of Ac_2O-pyridine (6 mL) and stirred at room temperature until the starting material was disappeared (48 h, TLC, 1:1 hexane-EtOAc). The reaction mixture was repeatedly coevapored with toluene (3 × 30 mL) and the residue purified by flash chromatography on silica gel (1:1 hexane-EtOAc) affording pure **15** (80.5 mg, 70% yield calculated from **6**) as a colourless syrup: R_f 0.67 (1:1 hexane-EtOAc); ^{1}H NMR (200 MHz, CD_3CN) δ: 7.38-7.26 (m, 10H, Ar-*H*), 5.80 (m, 1H, H-5), 5.60 (m, 2H, H-1, H-3), 4.91 (m, 1H, H-2), 4.58 (s, 4H, 2 × CH_2Ph), 3.74 (m, 2H, H-4, H-6), 2.07-2.05 (m, 9H, 3 × CH_3CO), 1.93 (s, 3H, CH_3CO); ^{13}C NMR (50 MHz, CD_3CN) δ: 171.6 (3 × C=O), 170.5 (C=O), 139.1 (2 × Ar-*C*), 129.3-128.5 (Ar-*C*H), 73.4 (C-4, C-6), 71.6 (2 × *C*H_2Ph), 68.2 (C-1, C-2, C-3), 67.9 (C-5), 21.2 (2 × *C*H_3CO), 21.1, 20.7 (2 × *C*H_3CO). Anal. Calcd for $C_{28}H_{32}O_{10}$: C, 63.63; H, 6.10. Found: C, 63.58; H, 6.07.

1,3-di-*O*-Benzyl-1L-*epi*-inositol (16).

To a solution of **11** (137 mg, 0.38 mmol) in MeOH (5 mL), $NaBH_4$ (59 mg, 4 equiv) was added at 0 °C. The mixture was stirred at 0 °C until the starting material was disappeared (30 min, TLC, 9:1 CH_2Cl_2-MeOH). The solution was neutralized with Amberlist 15, the solids were filtered off and the solution was concentrated under

diminished pressure. Purification of crude residue by flash chromatography on silica gel (8:2 $CHCl_3$-MeOH) afforded pure **16** (119 mg, 87% yield) as a colourless syrup: R_f 0.70 (8:2 $CHCl_3$-MeOH); $[\alpha]_D^{23}$ +18.6 (c 0.99, $CHCl_3$); ^{1}H NMR (250.13 MHz, CD_3CN-D_2O) δ: 7.41-7.24 (m, 10H, Ar-*H*), 4.65, 4.56 (AB system, 2H, $J_{A,B}$=11.8 Hz, C*H*$_2$Ph), 4.60, 4.55 (AB system, 2H, $J_{A,B}$=12.0 Hz, C*H*$_2$Ph), 4.28 (dd, 1H, $J_{1,2}$=2.8 Hz, $J_{2,3}$=3.2 Hz, H-2), 4.11 (dt, 1H, $J_{4,5}$=3.2 Hz, $J_{3,4}$=2.9 Hz, H-4), 3.87 (t, 1H, $J_{1,6}$=$J_{5,6}$=9.7 Hz, H-6), 3.27 (t, 1H, H-3), 3.24 (dd, 1H, H-5), 3.12 (dd, 1H, H-1); ^{13}C NMR (62.9 MHz, CD_3CN-D_2O) δ: 139.7, 139.5 (2 × Ar-*C*), 129.3-128.4 (Ar-*C*H), 80.8 (C-1), 75.1 (C-3), 73.5 (C-4), 73.1 (C-5), 72.2, 70.8 (2 × *C*H$_2$Ph), 71.1 (C-6), 70.4 (C-2). Anal. Calcd for $C_{20}H_{24}O_6$: C, 66.65; H, 6.71. Found: C, 66.68; H, 6.69.

The reduction of **6** (111 mg, 0.31 mmol) with $NaBH(OAc)_3$ (106 mg, 0.5 mmol) in CH_3CN (5 mL) and in presence of AcOH (0.4 mL) as reported from preparation of **14** gave, after chromatographic purification on silica gel (8:2 $CHCl_3$-MeOH), **16** (72.6 mg, 65% yield) having NMR parameters identical to those of the sample prepared above.

1,2,3,5-tetra-*O*-Acetyl-4,6-di-*O*-benzyl-1L-*epi*-inositol (16a).

A solution of **16** (52 mg, 0.144 mmol) in a 1:2 mixture of Ac_2O-pyridine (4 mL) was stirred at room temperature until the starting material was disappeared (15 h, TLC, 1:1 hexane-EtOAc). The reaction mixture was repeatedly coevapored with toluene (3 × 30 mL) and purification of residue by flash chromatography over silica gel (1:1 hexane-EtOAc) afforded pure **16a** (73.3 mg, 95% yield) as a colourless syrup: R_f 0.47 (1:1 hexane-EtOAc); $[\alpha]_D^{23}$ +27.5 (c 1.12, $CHCl_3$); ^{1}H NMR (250.13 MHz,

CD_3CN) δ: 7.43-7.24 (m, 10H, Ar-*H*), 5.91 (ddd, 1H, $J_{4,5}$=3.3 Hz, $J_{5,6}$=3.3 Hz, $J_{3,5}$=1.0 Hz, H-5), 5.65 (ddd, 1H, $J_{2,3}$=3.5 Hz, $J_{3,4}$=3.3 Hz, H-3), 5.49 (dd, 1H, $J_{1,2}$=10.6 Hz, $J_{1,6}$=10.2 Hz, H-1), 4.93 (dd, 1H, H-2), 4.64, 4.42 (AB system, 2H, $J_{A,B}$=11.7 Hz, CH_2Ph), 4.56 (s, 2H, CH_2Ph), 3.80 (t, 1H, H-4), 3.65 (dd, 1H, H-6), 2.10, 2.09, 1.99, 1.94 (4s, each 3H, 4 x CH_3CO); ^{13}C NMR (62.9 MHz, CD_3CN) δ: 171.3-171.2 (4 × C=O), 139.0, 138.9 (2 × Ar-*C*), 129.5-128.4 (Ar-*C*H), 76.1 (C-6), 72.9 (C-4), 72.4, 71.8 (2 × *C*H_2Ph), 70.2 (C-2), 69.7 (C-1), 69.4 (C-3), 68.3 (C-5), 21.1, 21.0, 20.8, 20.4 (4 × *C*H_3CO). Anal. Calcd for $C_{28}H_{32}O_{10}$: C, 63.63; H, 6.10. Found: C, 63.58; H, 6.07.

Hexa-*O*-acetyl-*allo*-inositolo (17a).

A solution of **7** (204 mg, 0.57 mmol) in EtOH (17 mL) was treated, at -78 °C, with $NaBH_4$ (155.6 mg, 4.0 mmol, 7 equiv). The solution was allowed to reach 15 °C. After 1.5 h (TLC, 1:3 hexane-EtOAc), the solution was cooled to 0 °C, water (0.40 mL) and a 5% HCl solution were slowly added to neutralise and the solvent removed under diminished pressure. Crystallization of crude residue (H_2O) afforded pure 2,6-di-*O*-benzyl-1D-*allo*-inositol (**17**) (188 mg, 91% yield) as a white solid: R_f 0.22 (1:3 hexane-EtOAc,); mp 120-124 °C (H_2O); ^{13}C NMR (50 MHz, CD_3OD,) δ 139.7, 139.1 (2 × Ar-*C*), 129.5-128.8 (Ar-*C*H), 74.5 (2 × CH), 71.6 (2 × CH), 69.6 (2 × CH); Anal. Calcd for $C_{20}H_{24}O_6$: C, 66.65; H, 6.71. Found: C, 66.68; H, 6.70.

A solution of **17** (188 mg, 0.52 mmol) in dry MeOH (16 mL) containing 10% Pd on charcoal (562 mg) was stirred at room temperature under H_2 (atmospheric pressure) until the starting compound was completely reacted (TLC, 7 h). The suspension was diluted with MeOH (16 mL), filtered over a short pad of Celite®, washed with MeOH. The combined organic phases were concentrated at diminished

pressure. The crude residue (120 mg) was dissolved in a 1:2 Ac_2O-Pyridine mixture (12 mL) at room temperature until starting material was disappeared (24 h, TLC, 1:1 hexane-EtOAc). The reaction mixture was repeatedly coevapored with toluene (3 × 30 mL). The residue was purified by crystallization (EtOH), affording pure **17a** (212 mg, 87% yield from **7**) as a white solid: R_f 0.37 (1:1 hexane-EtOAc); mp 140-142 °C (EtOH); Lit [7] mp 137-139 °C (EtOH); lit [8] mp 140-141 °C (MeOH); $[\alpha]_D^{23}$ 0.0 (c 1.1, $CHCl_3$); Lit [51] $[\alpha]_D$ 0.0 (c 0.35, $CHCl_3$); ^1H NMR (200 MHz, $CDCl_3$) δ: 5.48-5.37 (m, 4H), 5.29-5.22 (m, 2H), 2.09-2.01 (m, 18H, 6 × CH_3CO); ^{13}C NMR (50 MHz, $CDCl_3$) δ: 169.4-169.6 (6 × C=O), 67.1 (3 × CH), 67.5 (3 × CH), 20.6 (6 × CH_3CO). ^1H NMR data were in good agreement with those reported [7, 9].

1,5-di-O-Benzyl-1D-*chiro*-inositol (18).

To a solution of **7** (215 mg, 0.60 mmol) in CH_3CN (17 mL), AcOH (3.0 mL) and $NaBH(OAc)_3$ (1.22 g, 5.75 mmol) were consecutively added. The mixture was stirred at room temperature until the starting material was disappeared (1.15 h, TLC, 1:4 hexane-EtOAc). Excess of hydride was decomposed with a solution of aq $NaHSO_4$ (0.5 M, 20 mL). EtOAc (25 mL) was added, the organic phase was separated and the aq phase was further extracted with EtOAc (3 × 20 mL). The combined organic phases were collected, dried, filtered and concentrated under diminished pressure. Purification of residue by flash chromatography on silica gel (EtOAc) afforded pure **18** (97 mg, 45% yield) as a white solid: R_f 0.16 (9:1 $CHCl_3$-MeOH); mp 116-120 °C; $[\alpha]_D^{23}$ +9.41 (c 1.07, $CHCl_3$); ^1H NMR (200 MHz, $CDCl_3$,) δ: 7.35-7.18 (m, 10H, Ar-*H*), 4.67, 4.47 (AB system, 2H, J_{AB}=11.8 Hz, CH_2Ph), 4.64, 4.38 (AB system, 2H, J_{AB}=11.7 Hz, CH_2Ph), 3.95-3.84 (m, 3H, H-1, H-4, H-6); 3.83 (dd, 1H, $J_{2,3}$=9.0 Hz, $J_{1,2}$=3.6 Hz, H-2), 3.71 (t, 1H, $J_{2,3}$=$J_{3,4}$=9.0 Hz, H-3), 3.57 (dd, 1H, $J_{5,6}$=2.7 Hz, H-5),

3.38 (m, 4H, 4 × OH); ^{13}C NMR (50 MHz, $CDCl_3$) δ: 138.5, 137.9 (2 × Ar-*C*), 128.3-127.5 (Ar-*C*H), 79.3, 79.1 (C-1, C-5), 72.9, 73.5 (2 × *C*H_2Ph), 73.8, 72.3, 70.7, 67.7 (C-2, C-3, C-4, C-6). Anal. Calcd for $C_{20}H_{24}O_6$: C, 66.65; H, 6.71. Found: C, 66.71; H, 6.63.

D*-chiro-*Inositol (18a).

A solution of **18** (131 mg, 0.36 mmol) in dry MeOH (11 mL) containing 10% Pd on charcoal (43 mg) was stirred at room temperature under H_2 (atmospheric pressure) until the starting compound was completely reacted (TLC, 96 h). The suspension was diluted with MeOH (20 mL), filtered over a short pad of Celite®, washed with MeOH, and the combined organic phases were concentrated at diminished pressure. The crude residue was constituted (NMR) exclusively by **18a** (50.6 mg, 77% yield) as a white solid: mp 237-240 °C; $[\alpha]_D^{23}$ +62.3 (c 0.85, H_2O); Lit. [10] mp 238-242 °C (EtOH); $[\alpha]_D$ +63.2 (c 1.0, H_2O); ^{1}H NMR (200 MHz, D_2O) δ: 3.76 (m, 2H), 3.48 (m, 2H), 3.33 (m, 2H); ^{13}C NMR (50 MHz, D_2O) δ: 72.8 (C-3, C-4), 71.7 (C-1, C-6), 70.5 (C-2, C-5). NMR data were in good agreement with those reported [6, 10].

1,3-di-O-Benzyl-*myo*-inositol (19).

To a solution of **11** (232 mg, 0.65 mmol) in CH_3CN (32 mL), AcOH (2.5 mL) and $NaBH(OAc)_3$ (255 mg, 1.2 mmol) were consecutively added and the mixture was stirred at room temperature. After 1 h, further $NaBH(OAc)_3$ (270 mg, 1.27 mmol) was added and the solution stirred until the starting material was disappeared (TLC, 8:2 $CHCl_3$-MeOH). After 2 h and 20 min, the solvent was removed under diminished pressure and residue was purified by flash chromatography on silica gel (9:1 $CHCl_3$-MeOH) affording pure **19** (163 mg, 70% yield) as a syrup: R_f 0.48 (8:2 $CHCl_3$-MeOH); ^{1}H NMR (250.13 MHz, CD_3CN-D_2O) δ: 7.60-7.20 (m, 10H, Ar-*H*), 4.63, 4.53

(2 × AB system, each 2H, $J_{A,B}$=11.9 Hz, 2 × C*H*$_2$Ph), 4.19 (t, 1H, $J_{1,2}$=$J_{2,3}$=2.7 Hz, H-2), 3.64 (bt, 2H, spl. ≅ 9.4 Hz, H-4, H-6), 3.15 (dd, 2H, H-1, H-3), 3.12 (t, 1H, $J_{4,5}$=$J_{5,6}$=9.5 Hz, H-5); ^{13}C NMR (62.9 MHz, CD_3CN-D_2O) δ: 139.1 (2 × Ar-*C*), 129.3-128.6 (Ar-*C*H), 79.8 (C-1, C-3), 75.3 (C-5), 72.5 (C-4, C-6), 72.3 (2 × *C*H$_2$Ph), 68.6 (C-2). Anal. Calcd for $C_{20}H_{24}O_6$: C, 66.65; H, 6.71. Found: C, 66.62; H, 6.75.

5-*O*-Allyl-3-O-methyl-1-*O*-naphthalenylmethyl-1D-*chiro*-inositol (22).

To a solution of inosose **21** (210 mg, 0.56 mmol) in CH_3CN (23 mL), AcOH (2.7 mL) and NaBH(OAc)$_3$ (1.14 g, 5.36 mmol) were consecutively added and the mixture was stirred at room temperature until the starting material was disappeared (2 h, TLC, EtOAc). Excess of hydride was decomposed with a solution of aq $NaHSO_4$ (0.5 M) and the reaction mixture was repeatedly coevaporated with toluene (3 × 20 mL). The residue was partitioned between brine (20 mL) and EtOAc (20 mL), the organic phase was separated and the aq layer extracted with EtOAc (3 x 30 mL). The combined organic phases were collected, dried, filtered and concentrated under diminished pressure. Purification of the residue by flash chromatography (EtOAc) afforded **22** (151.6 mg, 72% yield) as a white foam: R_f 0.24 (EtOAc); $[\alpha]_D^{23}$ +26.6 (*c* 0.99, $CHCl_3$); ^{1}H NMR (CD_3CN, 250.13 MHz,) δ: 7.87 (m, 4H, Ar-*H*), 7.52 (m, 3H, Ar-*H*), 5.92 (ddt, 1H, J_{trans}=17.3 Hz, J_{cis}=10.4 Hz, *J*=5.7 Hz, C*H*=), 5.30 (dq, 1H, J_{trans}=17.3 Hz, *J*=1.6 Hz, C*H$_2$*=), 5.13 (dq, 1H, J_{cis}=10.4 Hz, *J*=1.3 Hz, C*H$_2$*=), 4.86, 4.75 (AB system, 2H, J_{AB}=12.4 Hz, C*H$_2$*Nap), 4.08 (ddd, 1H, $J_{1,6}$=3.8 Hz, $J_{5,6}$=3.0 Hz, $J_{6,OH}$=2.9 Hz, H-6), 4.04 (m, 2H, C*H$_2$*O), 3.78 (dd, 1H, $J_{1,2}$=3.5 Hz, H-1), 3.70 (ddd, 1H, $J_{2,3}$=9.6 Hz, $J_{2,OH}$=6.7 Hz, H-2), 3.58 (dt, 1H, $J_{3,4}$=9.5 Hz, $J_{4,OH}$=3.0 Hz, H-4), 3.54 (s, 3H, C*H$_3$*O), 3.39 (dd, 1H, H-5), 3.15 (dd, 1H, H-3), 3.19 (d, 1H, OH-4), 3.14 (d, 1H, OH-6), 3.13 (d, 1H, OH-2); ^{13}C NMR (CD_3CN, 62.9 MHz,) δ: 137.5, 134.2,

133.8 (3 × Ar-*C*), 136.6 (*C*H=), 128.9-126.9 (Ar-*C*H), 117.1 (CH_2=), 84.8 (C-3), 80.9 (C-1), 80.2 (C-5), 74.2 (CH_2Nap), 73.0 (C-4), 71.6 (CH_2O), 71.5 (C-2), 67.2 (C-6), 60.8 (CH_3O). Anal. Calcd for $C_{21}H_{26}O_6$: C, 67.36; H, 7.00. Found: C, 67.38; H, 6.98.

References

1. Catelani, G.; D'Andrea, F.; Guazzelli, L.; Pistarà V. *Carbohydr. Res.* **2009,** 344, 717-724; doi:10.1016/j.carres.2009.01.014
2. Pistarà, V.; Barili, P. L.; Catelani, G.; Corsaro, A.; D'Andrea, F.; Fisichella, S. *Tetrahedron Lett*. **2000**, *41*, 3253-3256; doi:10.1016/S0040-4039(00)00360-9
3. Guazzelli, L.; Catelani, G.; D'Andrea, F. *Carbohydr. Res.* **2010,** 345, 369-376; doi:10.1016/j.carres.2009.11.027
4. Hudlicky, T.; Mandel, M.; Rouden, J.; Lee, R. S.; Bachmann, B.; Dudding, T.; Yost, K.; Merola, J. S. *J. Chem. Soc., Perkin Trans. 1* **1994**, 1553-1567; doi: 10.1039/P1994000155
5. Takahashi, H.; Kittaka, H.; Ikegami, S. *J. Org. Chem.* **2001**, *66*, 2705-2716; doi: 10.1021/jo001575h
6. Angyal, S. J.; Odier, L. *Carbohydr. Res.* **1982**, *100*, 43-54; doi:10.1016/S0008-6215(00)81024-1
7. Jagdhane, R. C.; Shashidhar, M. S. *Eur. J. Org. Chem.* **2010**, 2945-2953; doi: 10.1002/ejoc.201000009
8. Motherwell, W. B.; Williams, A. S. *Angew. Chem., Int. Ed.* **1995**, *34*, 2031-2033; doi: 10.1002/anie.199520311
9. Yong, J. L.; Kyunghoon, L.; Sea, I. J., Heung, B. J.; Kwan, S. K. *Tetrahedron* **2005**, *61*, 1987-2001; doi:10.1016/j.tet.2005.01.003
10. Mandel, M.; Hudlicky, T. *J. Org. Chem.* **1993**, *58*, 2331-2333; doi: 10.1021/jo00060a061

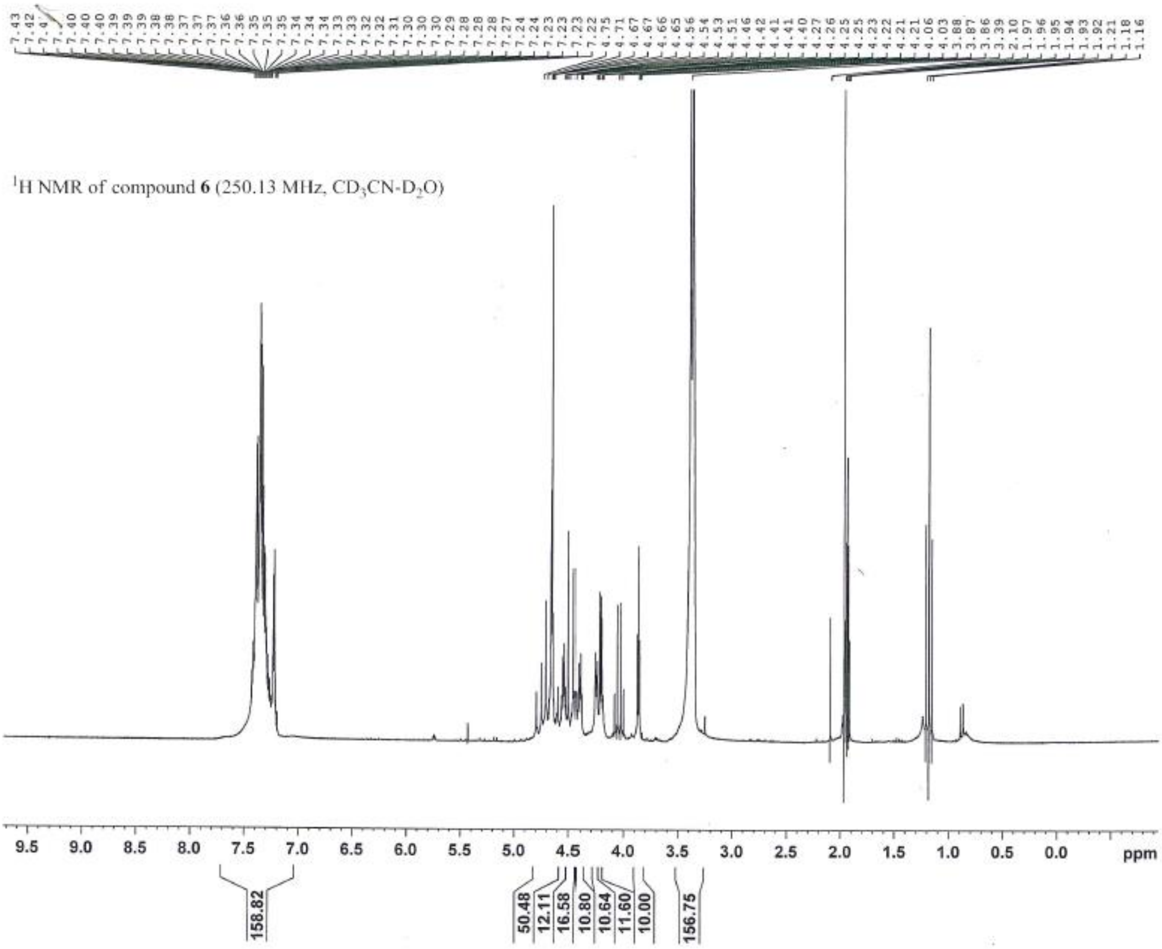

^{1}H NMR of compound **6** (250.13 MHz, CD_3CN-D_2O)

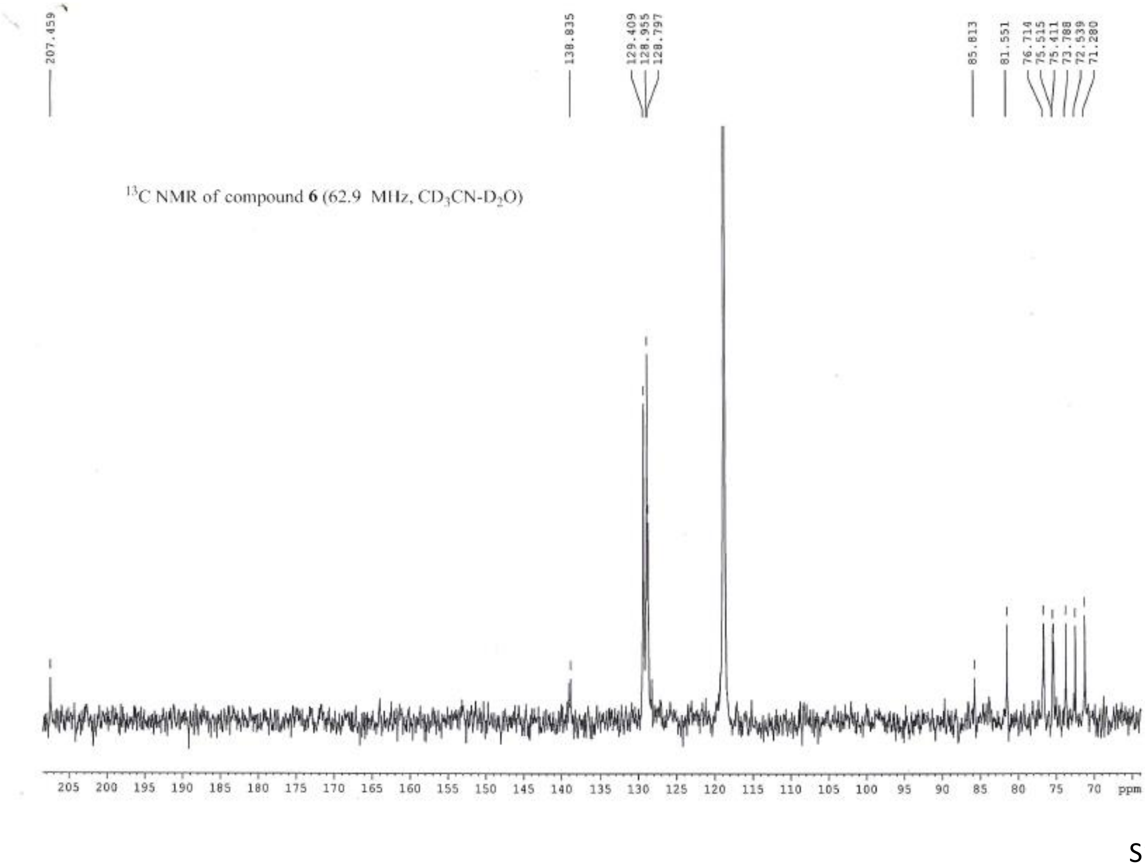

^{13}C NMR of compound **6** (62.9 MHz, CD_3CN-D_2O)

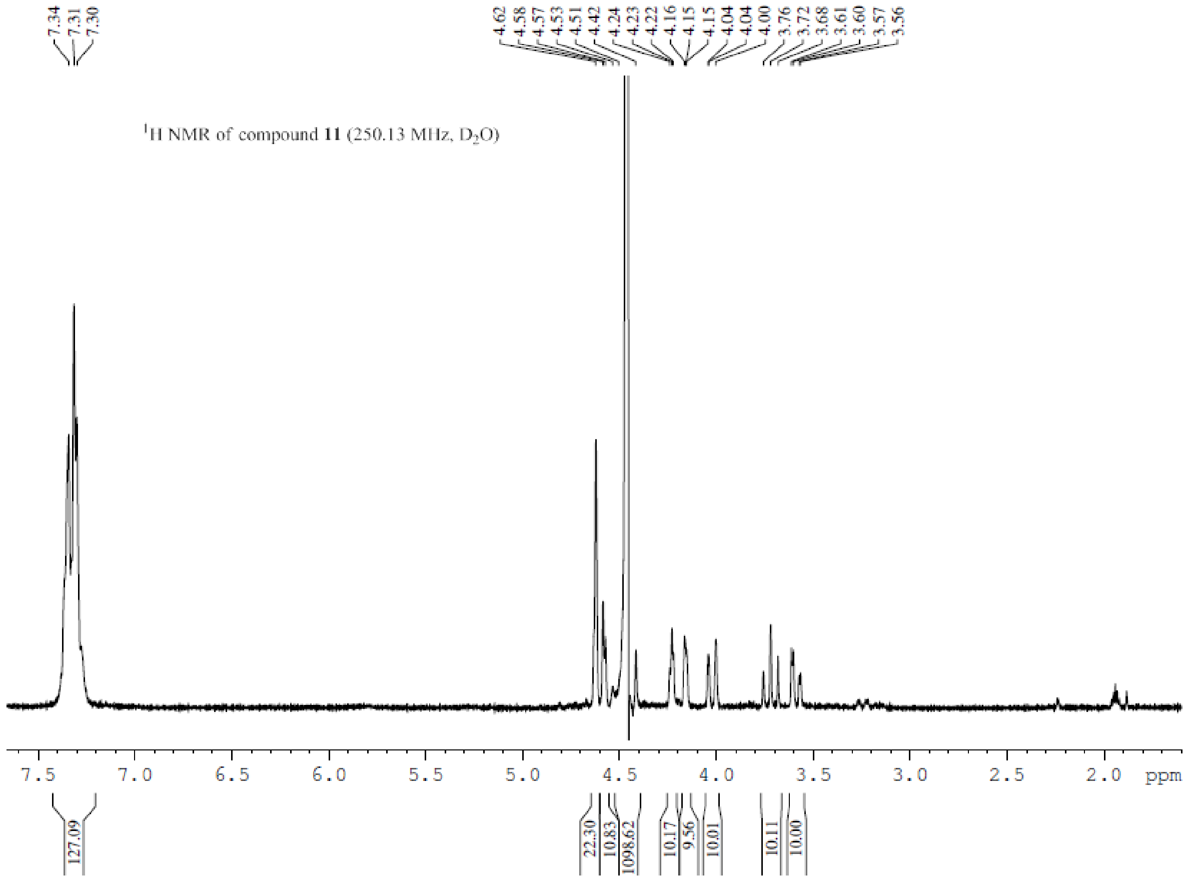
7.34
7.31
7.30
4.62
4.58
4.57
4.53
4.51
4.42
4.24
4.23
4.22
4.16
4.15
4.15
4.04
4.04
4.00
3.76
3.72
3.68
3.61
3.60
3.57
3.56
^{1}H NMR of compound **11** (250.13 MHz, D_2O)
7.5
7.0
6.5
6.0
5.5
5.0
4.5
4.0
3.5
3.0
2.5
2.0
ppm
127.09
22.30
10.83
1098.62
10.17
9.56
10.01
10.11
10.00

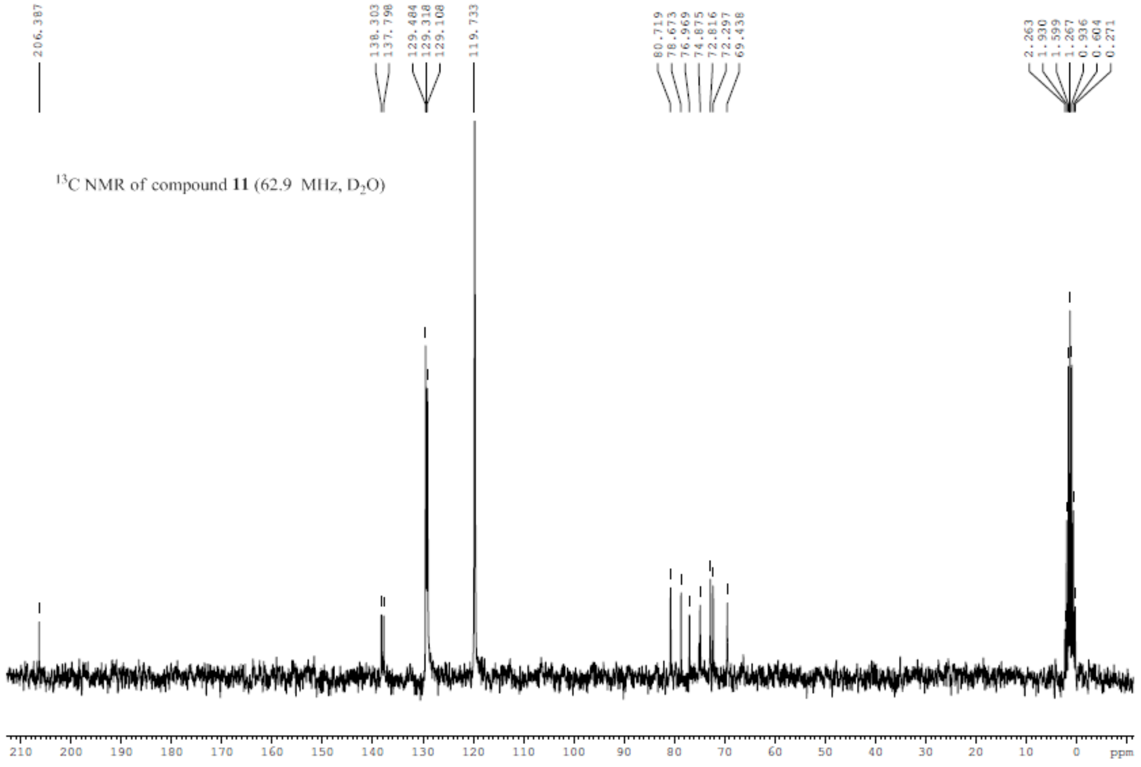
206.387
138.303
137.798
129.484
129.318
129.108
119.733
80.719
78.673
76.969
74.875
72.816
72.297
69.438
2.263
1.930
1.599
1.267
0.936
0.604
0.271
^{13}C NMR of compound **11** (62.9 MHz, D_2O)
210
200
190
180
170
160
150
140
130
120
110
100
90
80
70
60
50
40
30
20
10
0
ppm

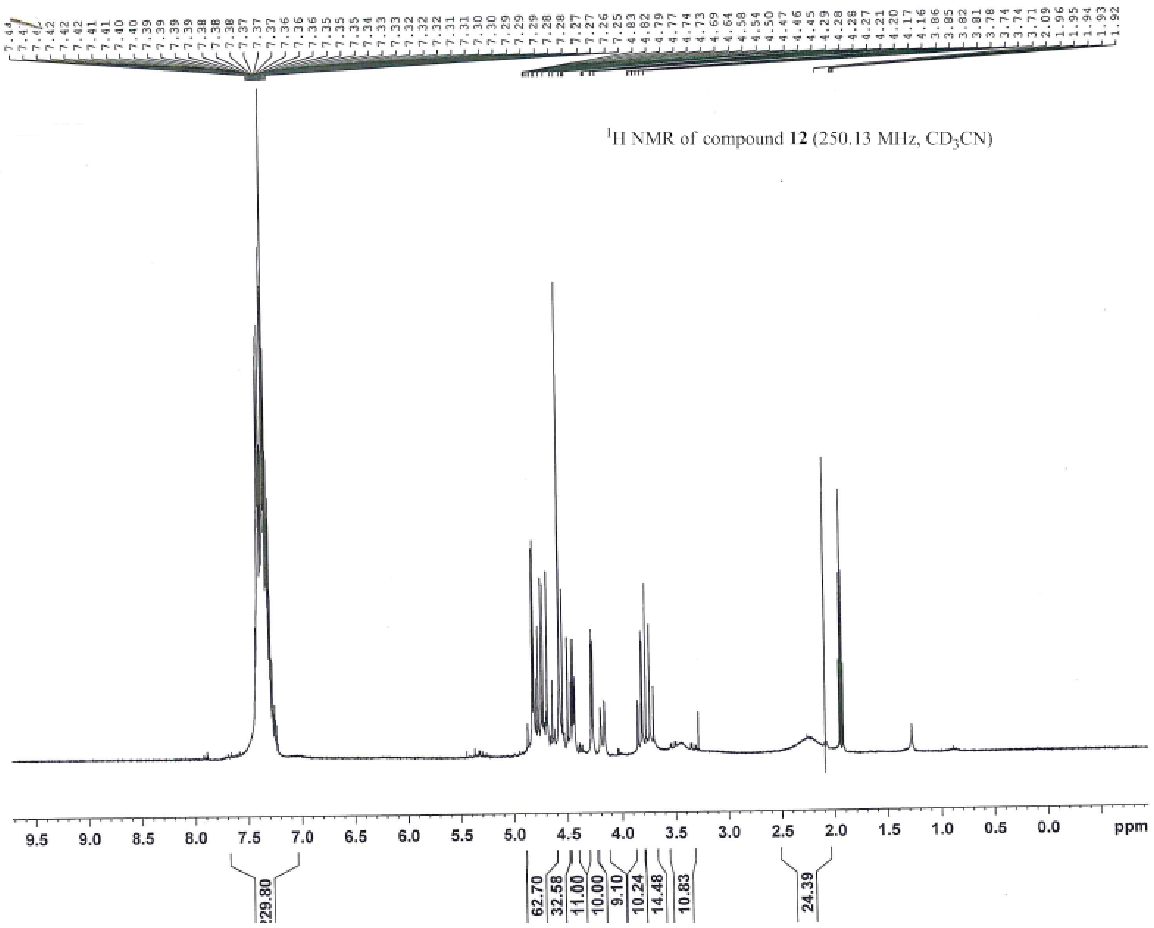

^{1}H NMR of compound 12 (250.13 MHz, CD3CN)

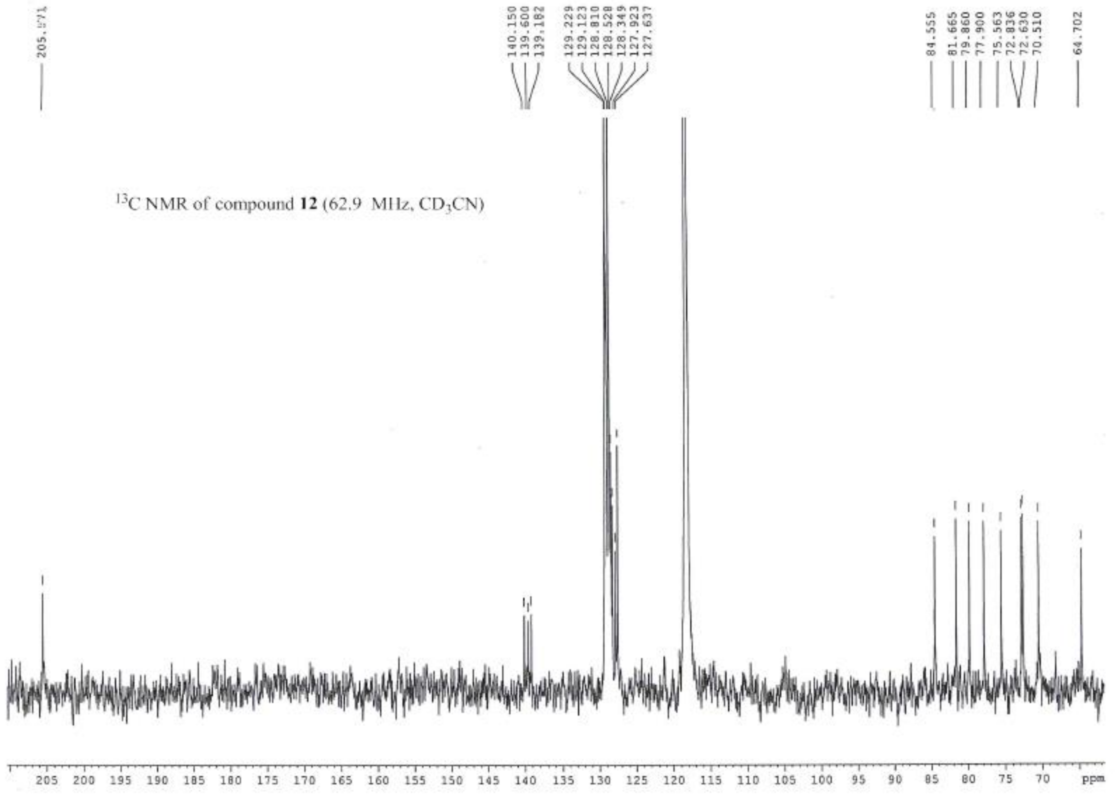

^{13}C NMR of compound 12 (62.9 MHz, CD3CN)

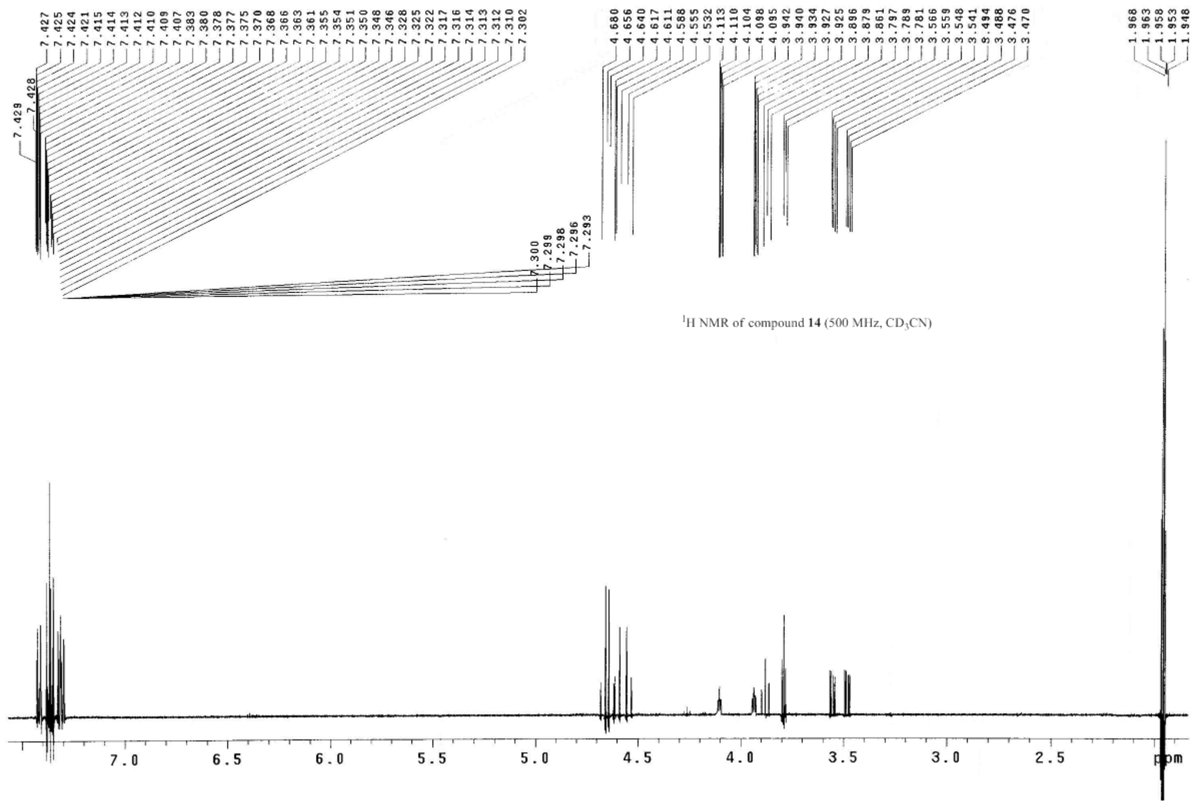

^{1}H NMR of compound **14** (500 MHz, CD_3CN)

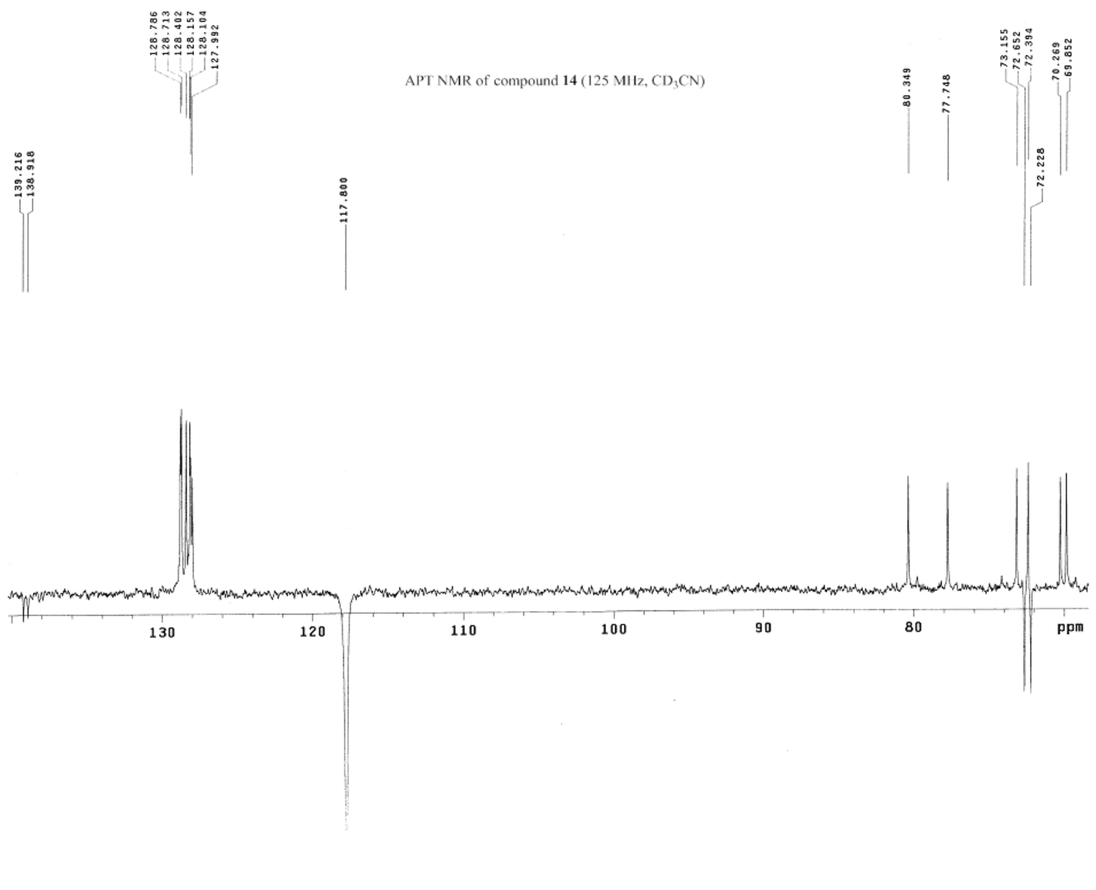

APT NMR of compound **14** (125 MHz, CD_3CN)

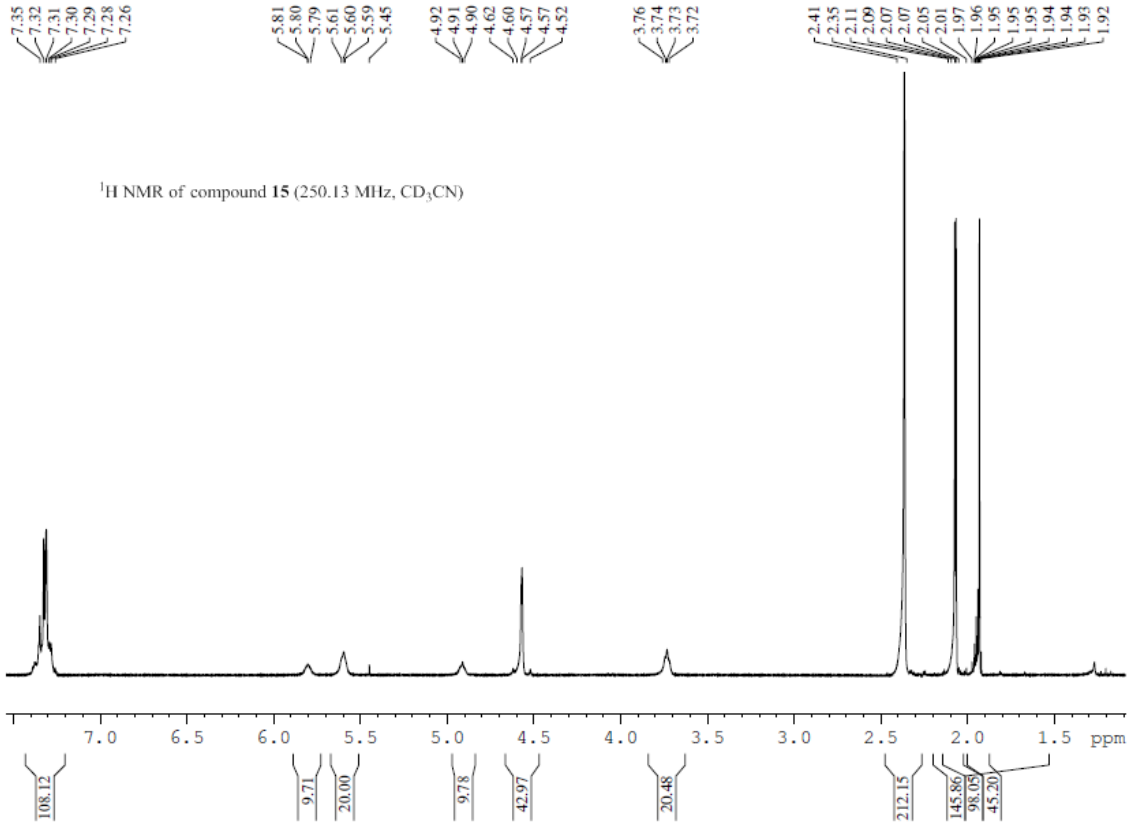

^{1}H NMR of compound **15** (250.13 MHz, CD_3CN)

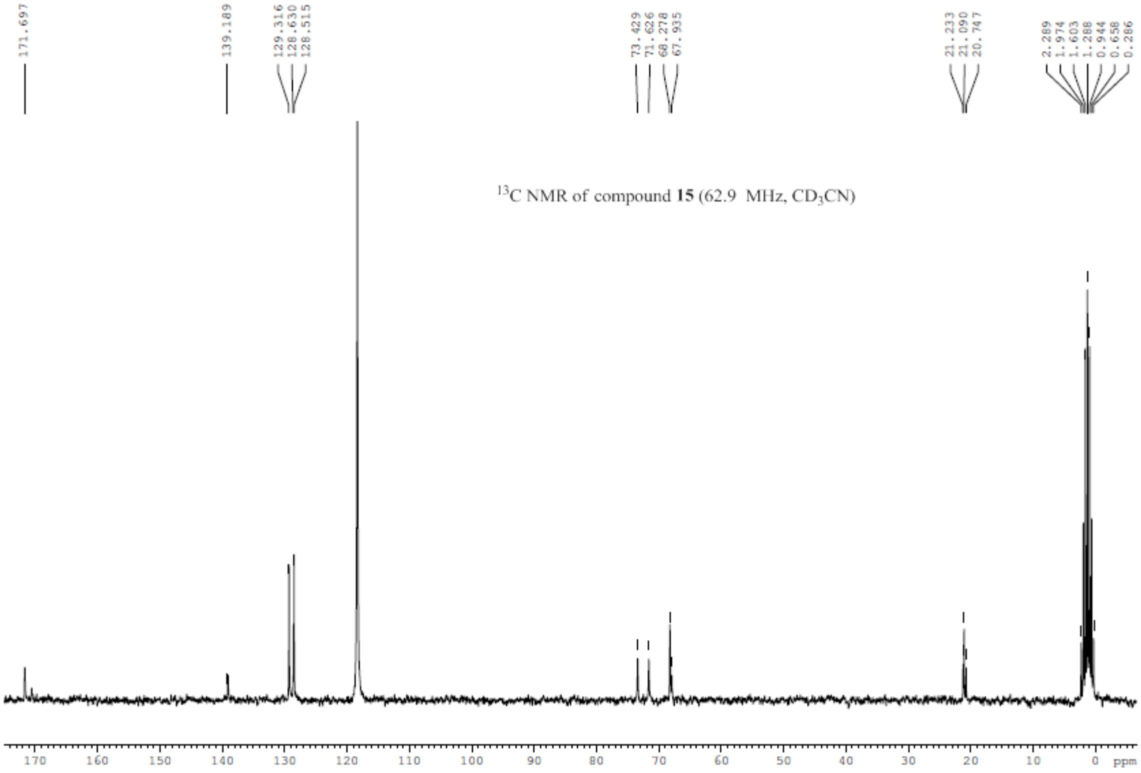

^{13}C NMR of compound **15** (62.9 MHz, CD_3CN)

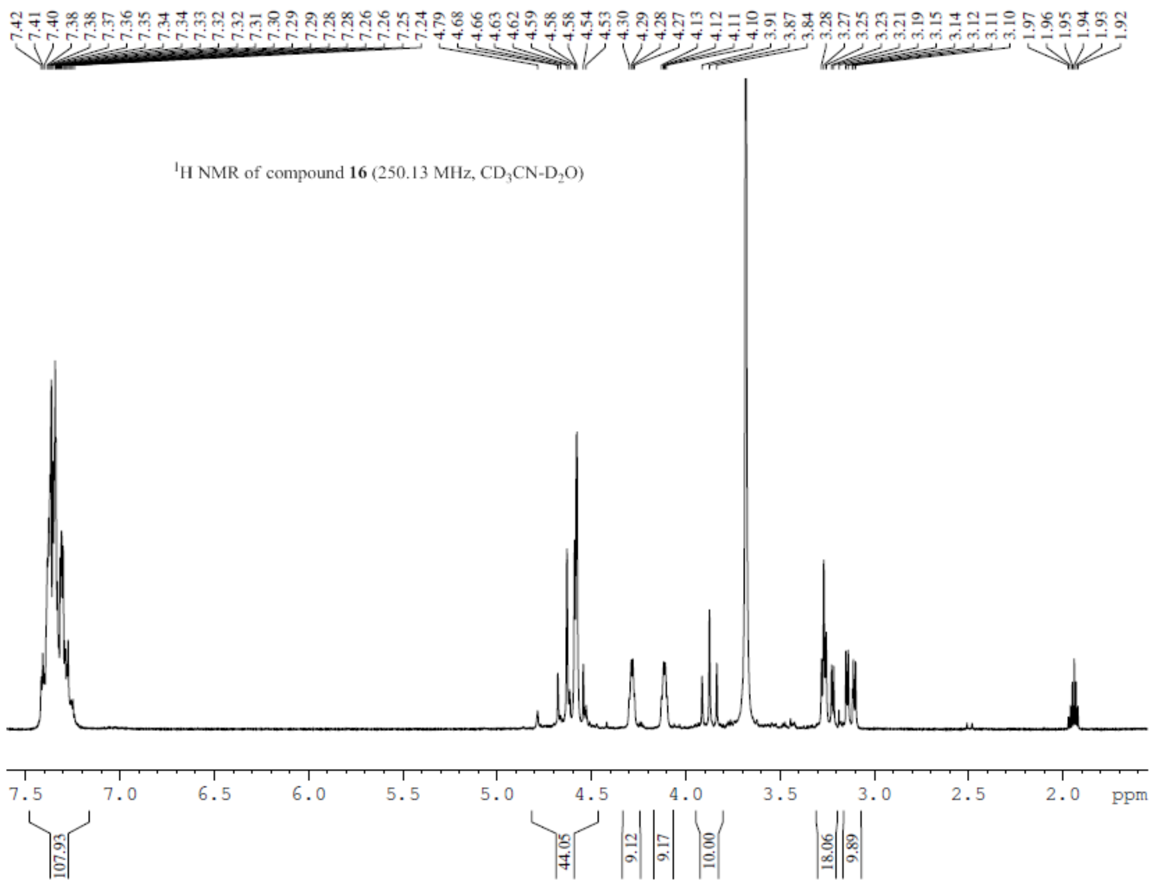
^{1}H NMR of compound **16** (250.13 MHz, CD_3CN-D_2O)
ppm

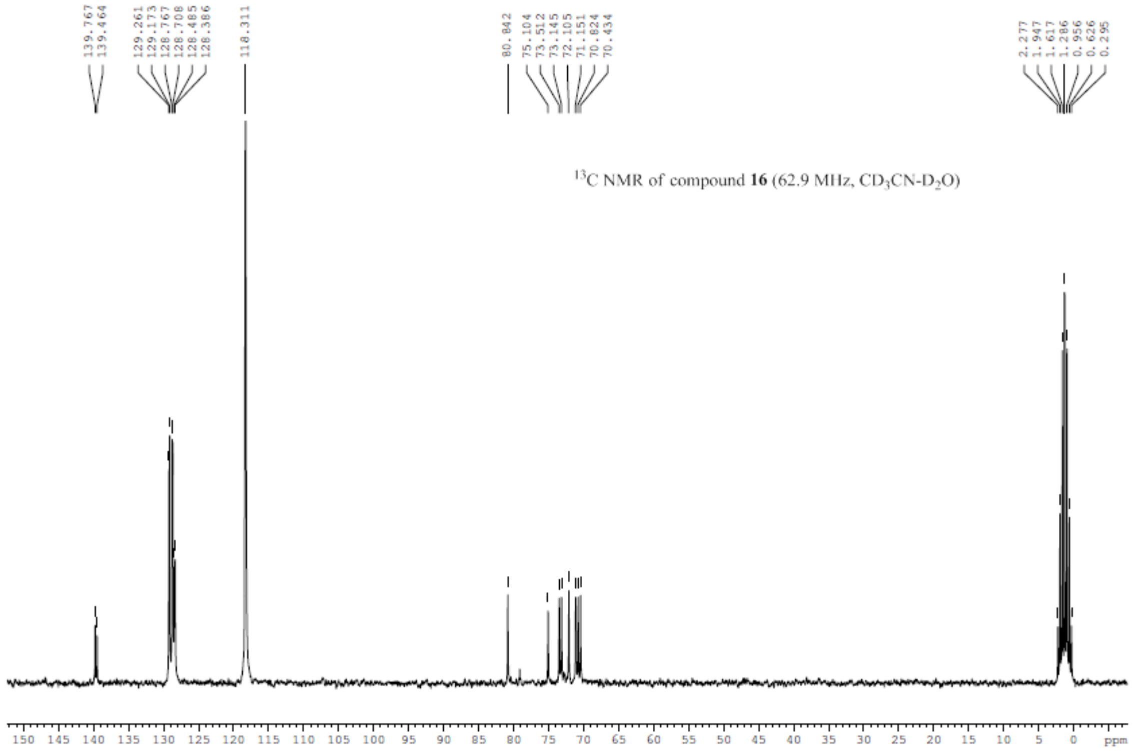
^{13}C NMR of compound **16** (62.9 MHz, CD_3CN-D_2O)
ppm

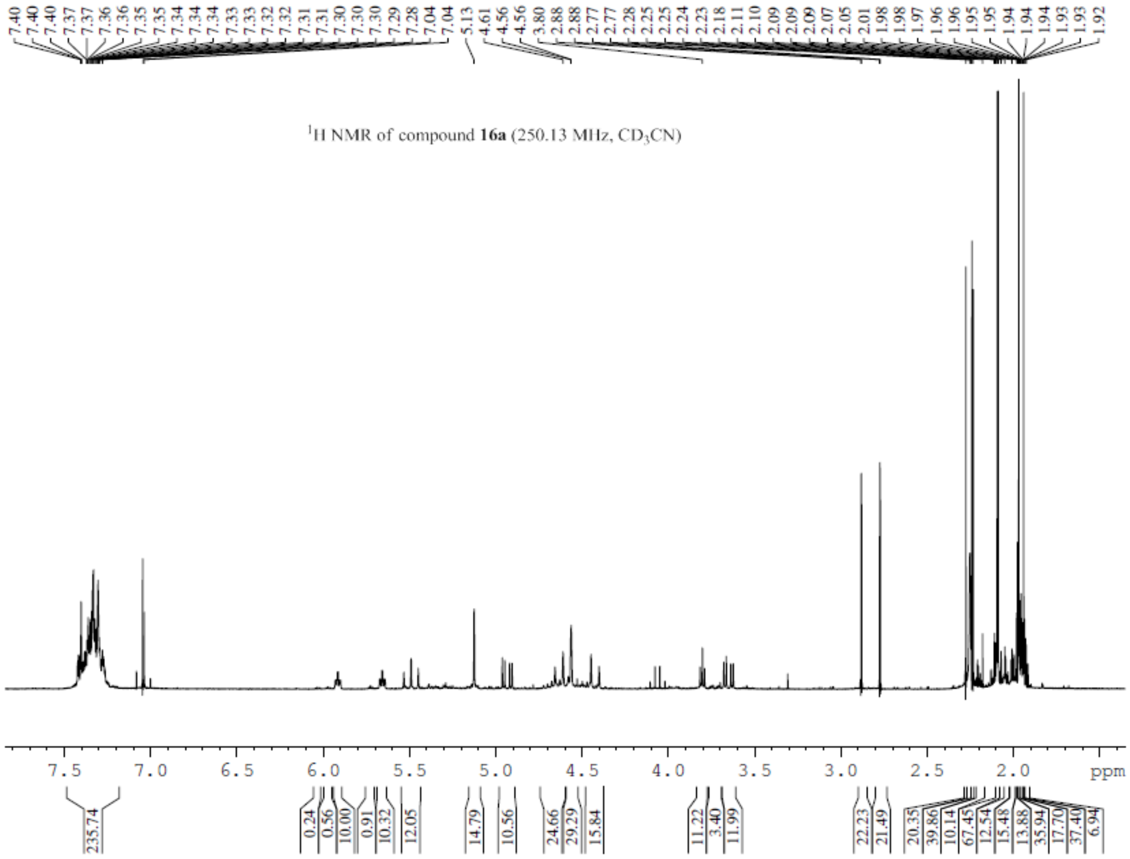
^{1}H NMR of compound **16a** (250.13 MHz, CD_3CN)

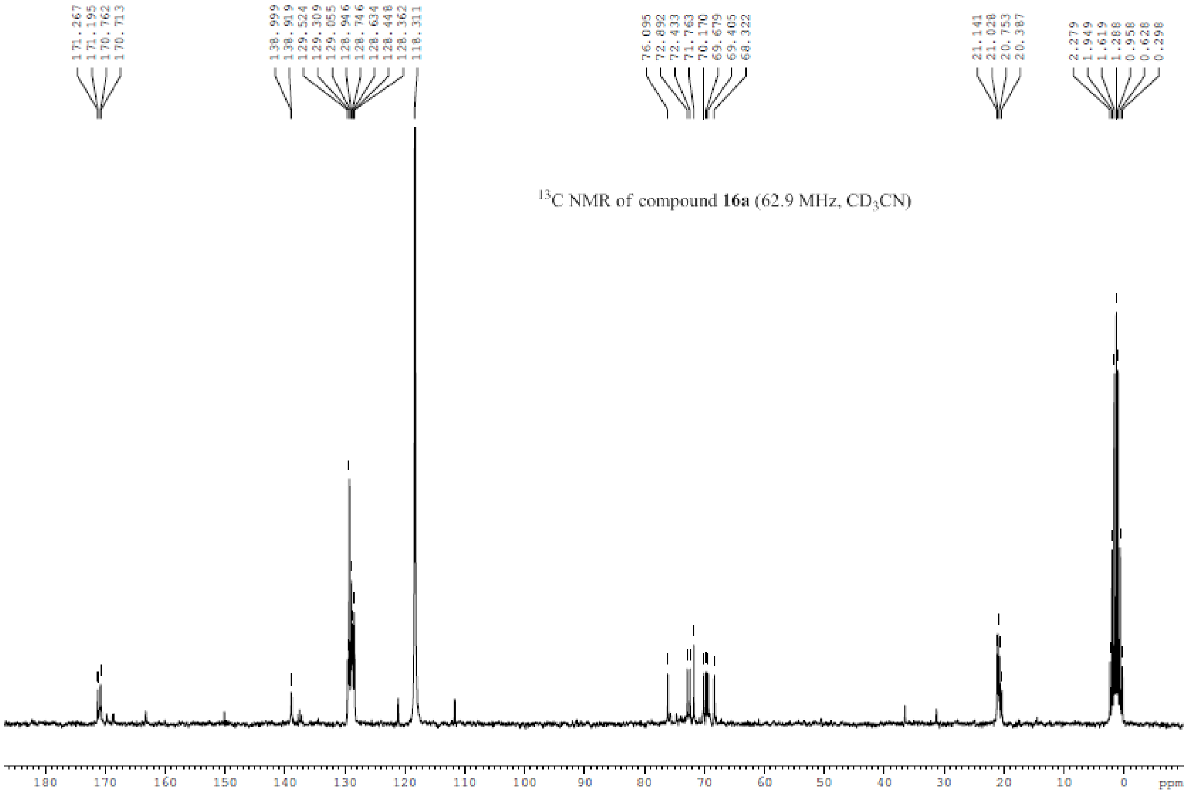
^{13}C NMR of compound **16a** (62.9 MHz, CD_3CN)

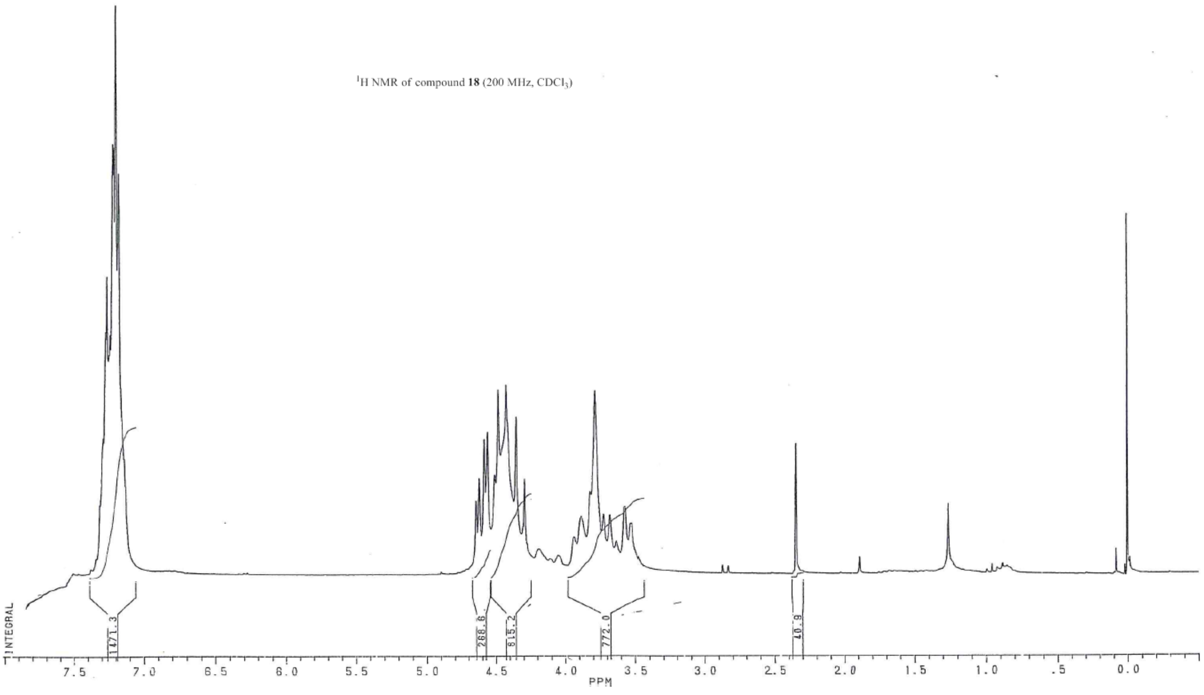

^{1}H NMR of compound **18** (200 MHz, CDCl$_3$)
INTEGRAL
PPM

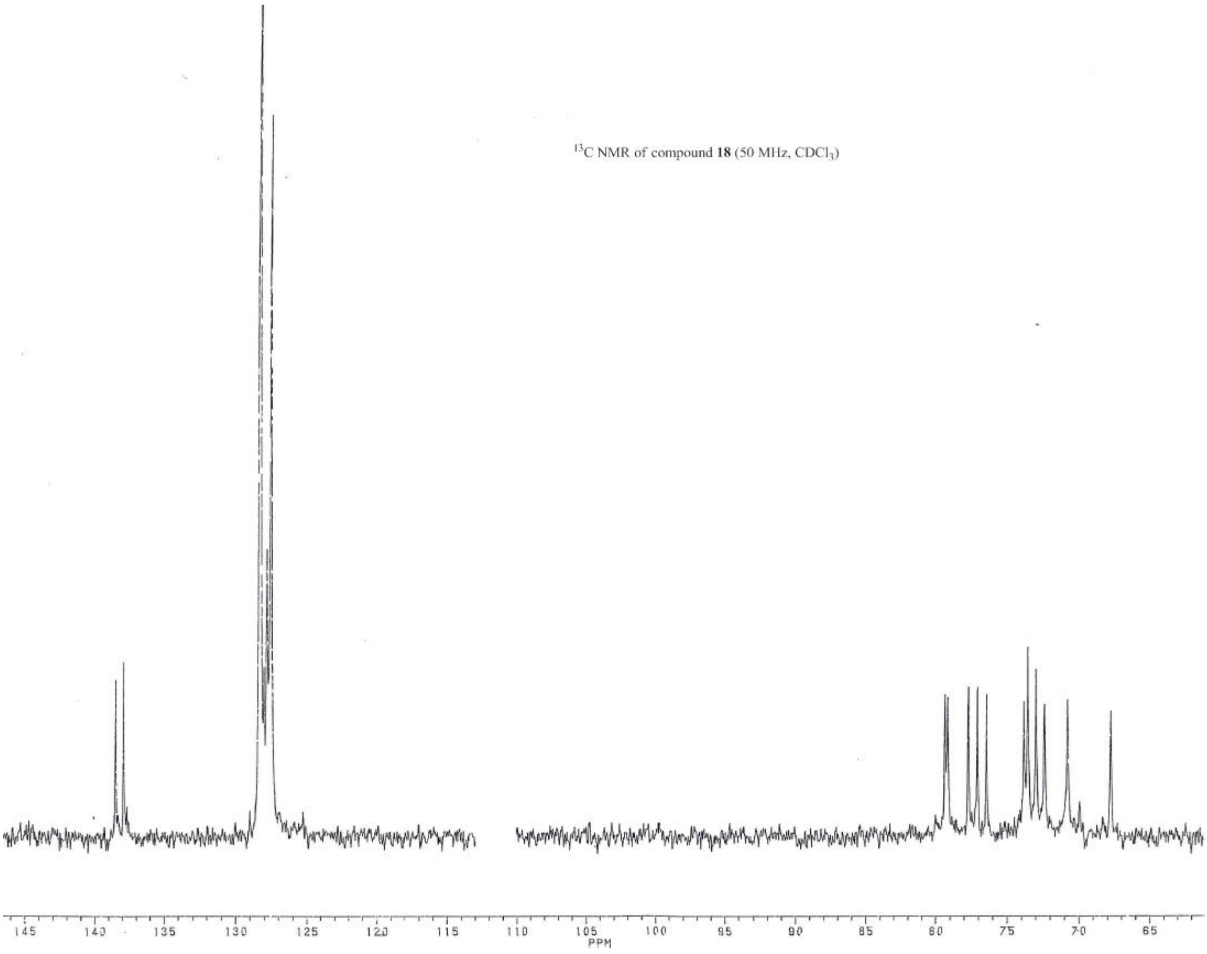

^{13}C NMR of compound **18** (50 MHz, CDCl$_3$)
PPM

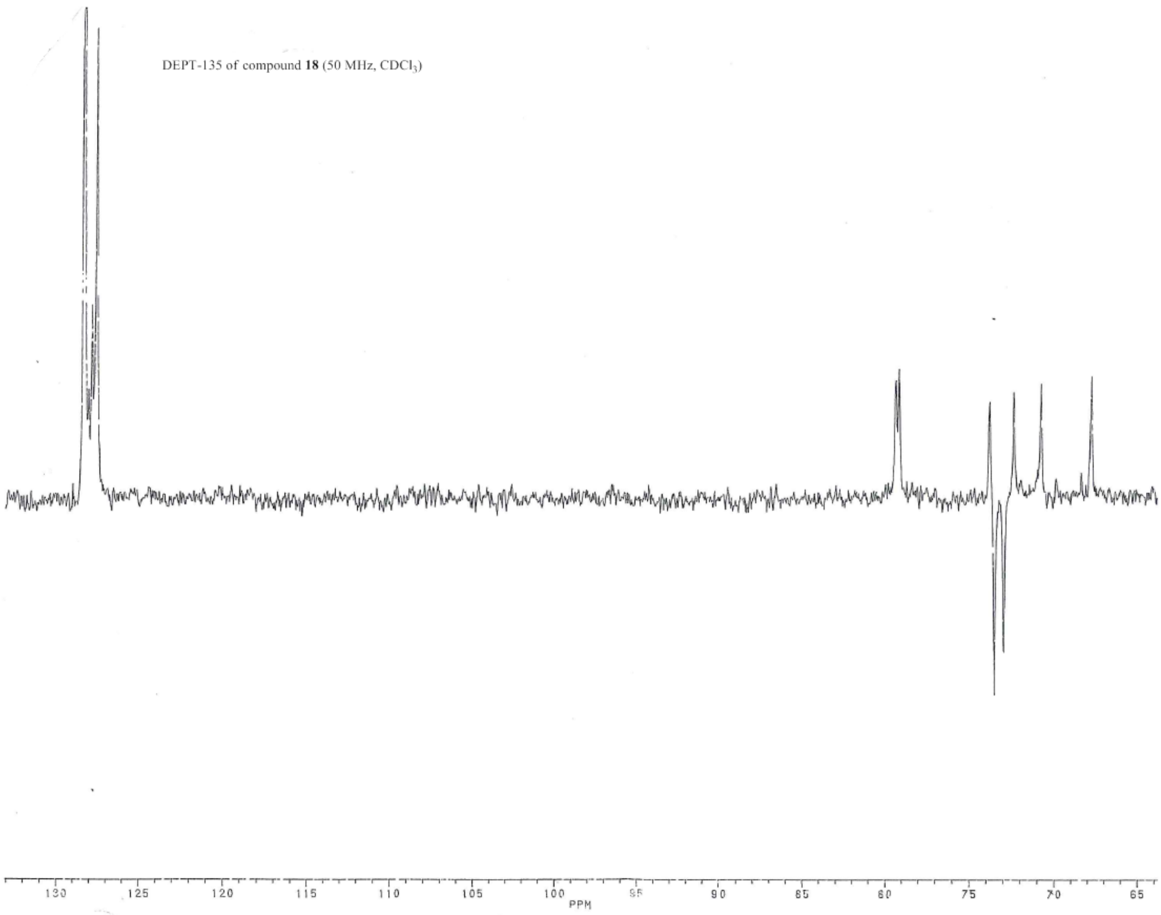

DEPT-135 of compound **18** (50 MHz, $CDCl_3$)

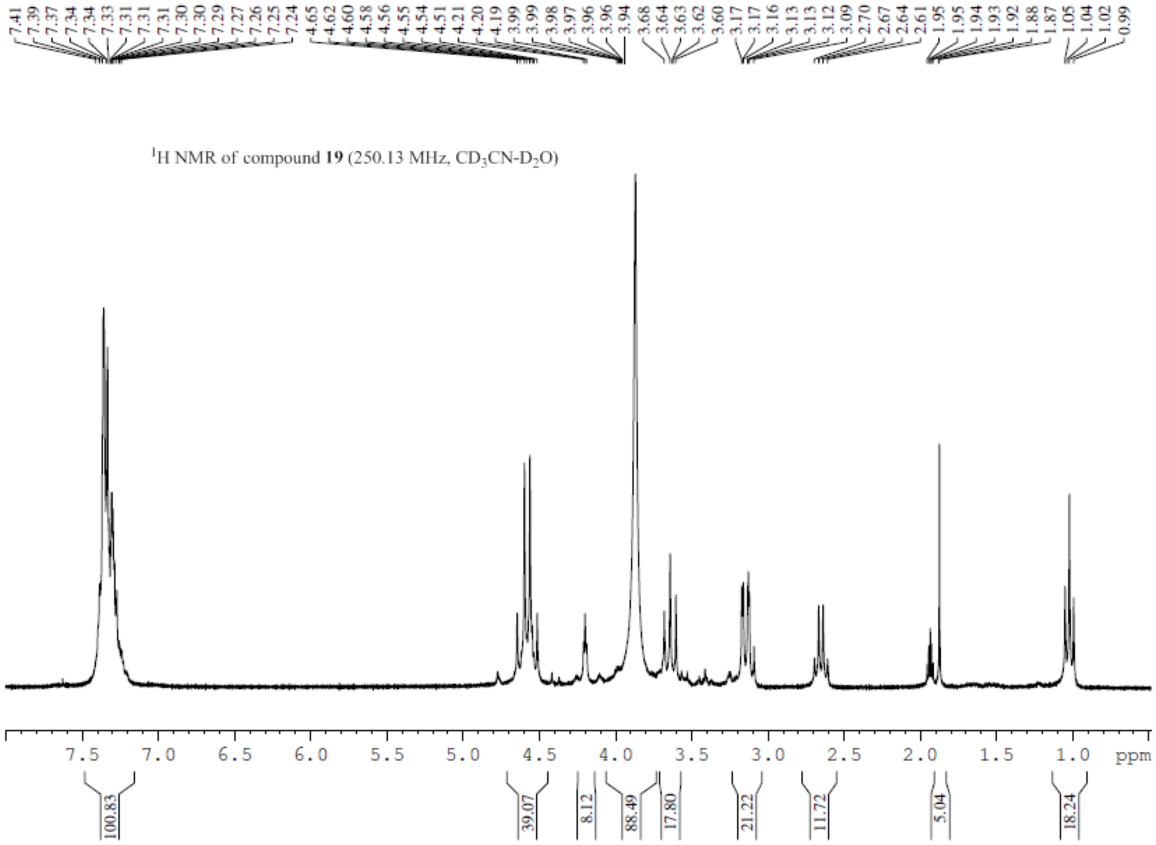

^{1}H NMR of compound **19** (250.13 MHz, CD_3CN-D_2O)

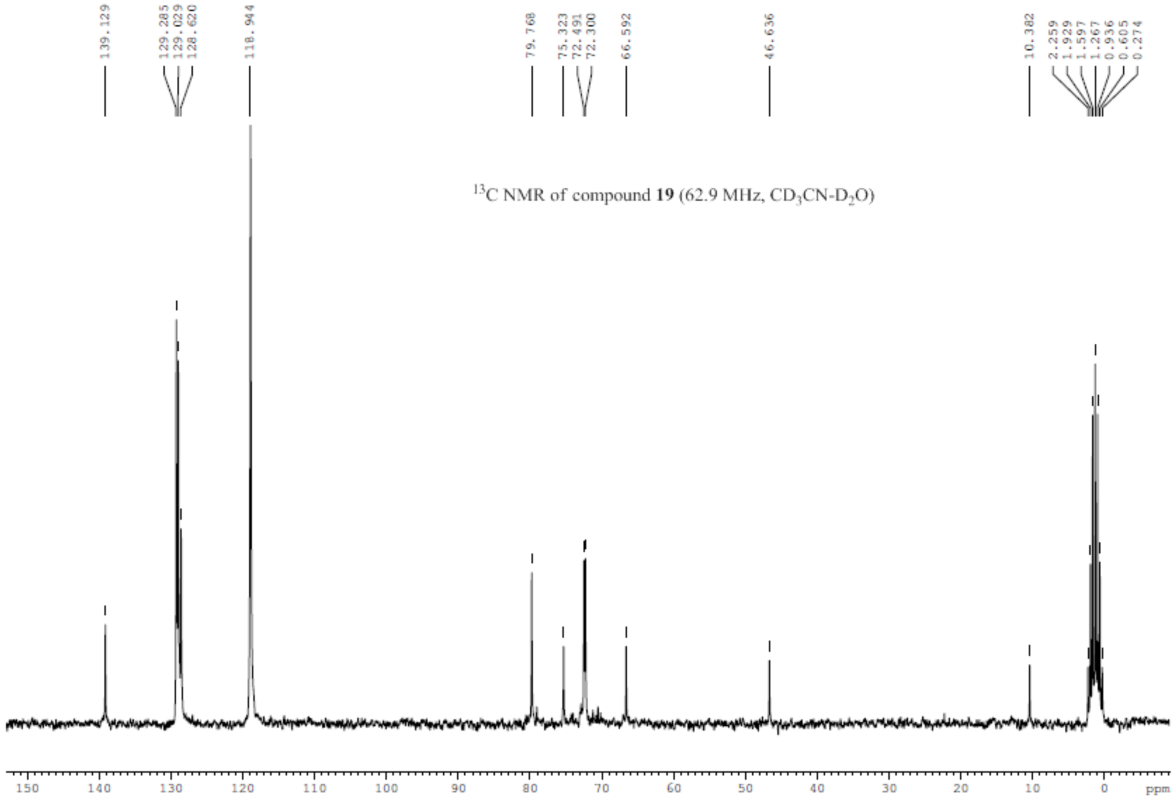

^{13}C NMR of compound **19** (62.9 MHz, CD_3CN-D_2O)

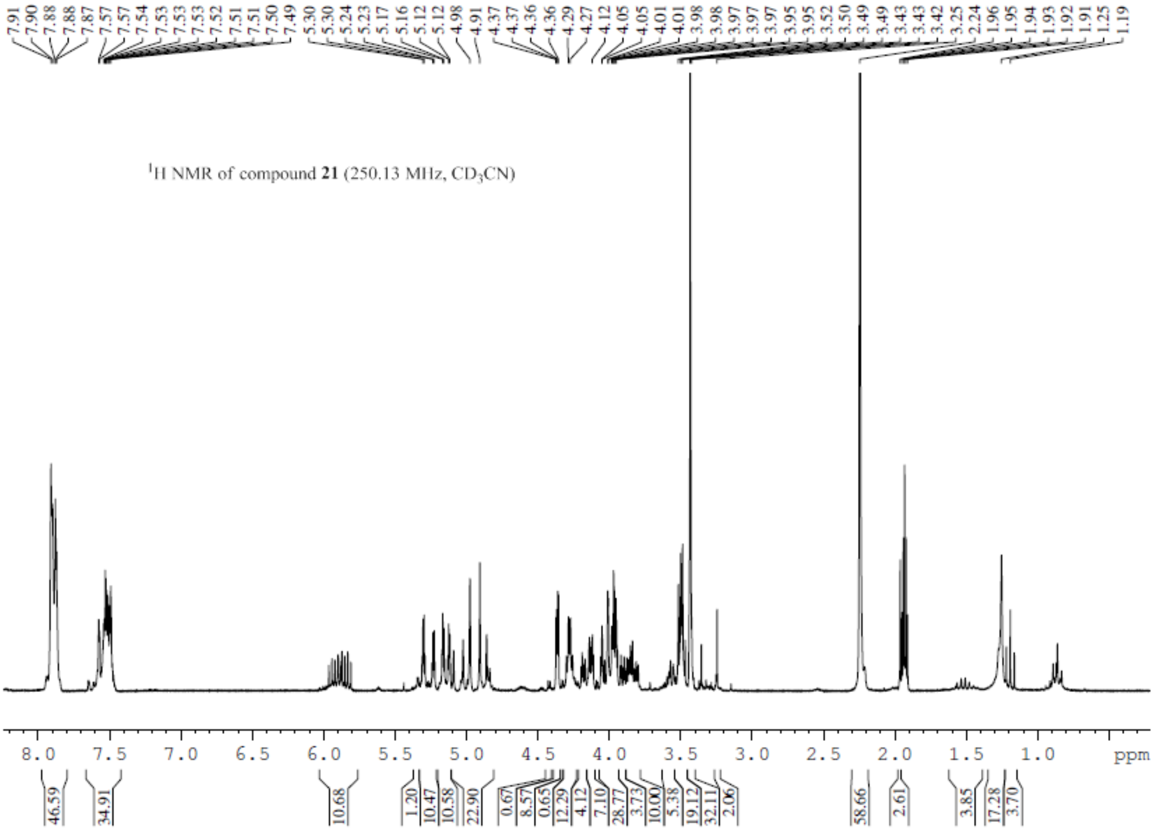

^{1}H NMR of compound **21** (250.13 MHz, CD_3CN)

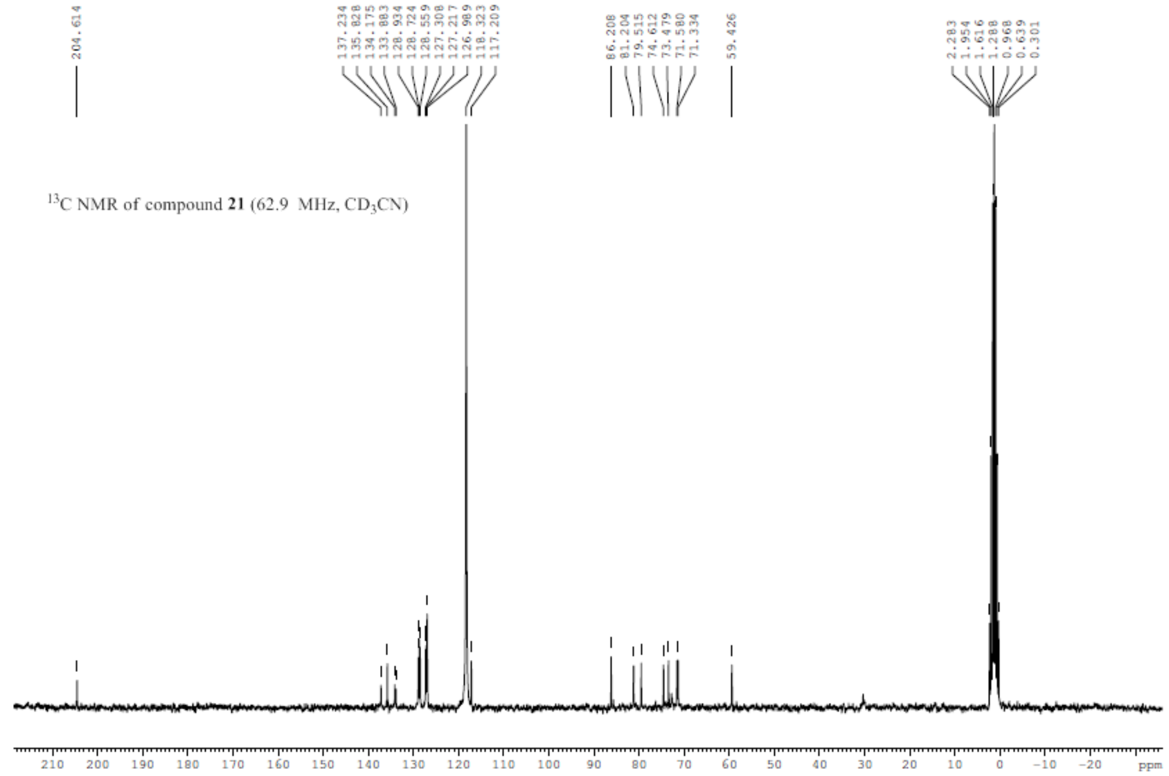
^{13}C NMR of compound **21** (62.9 MHz, CD_3CN)

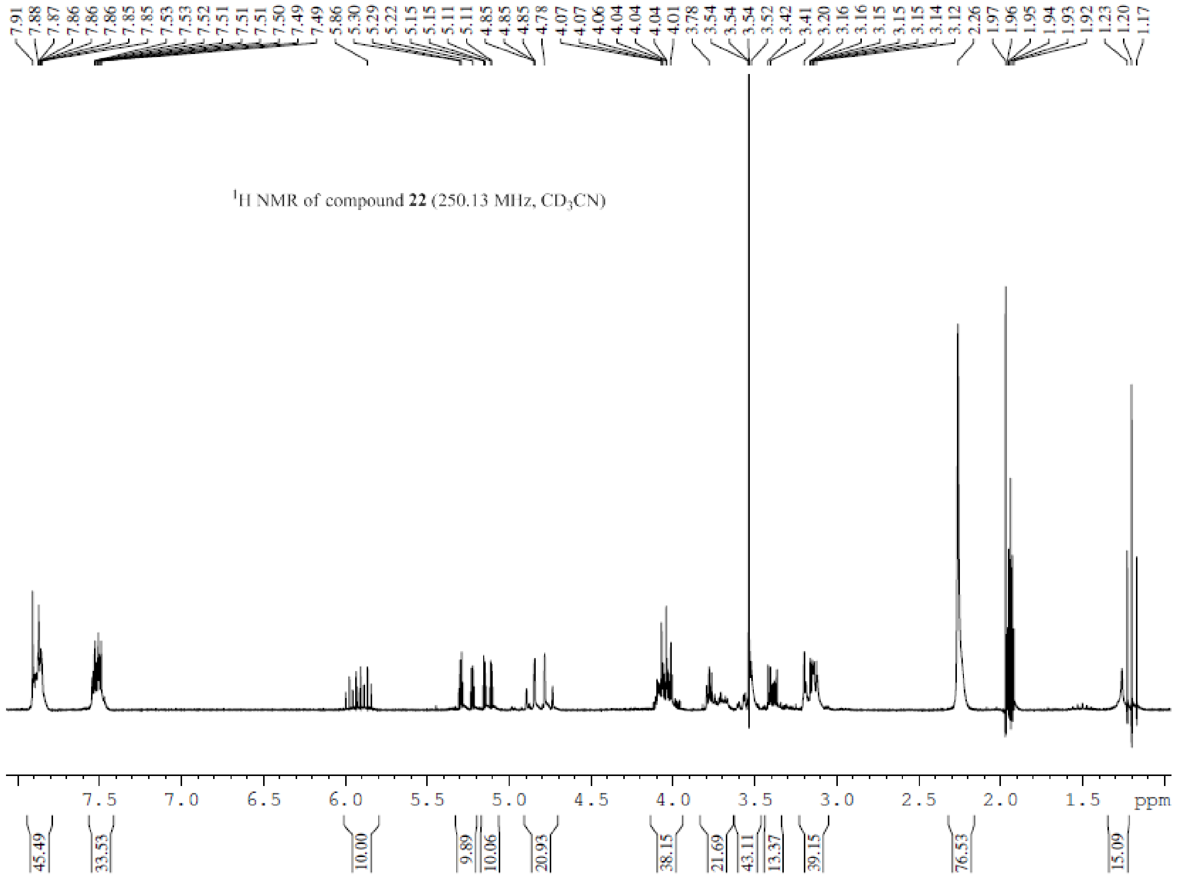
^{1}H NMR of compound **22** (250.13 MHz, CD_3CN)

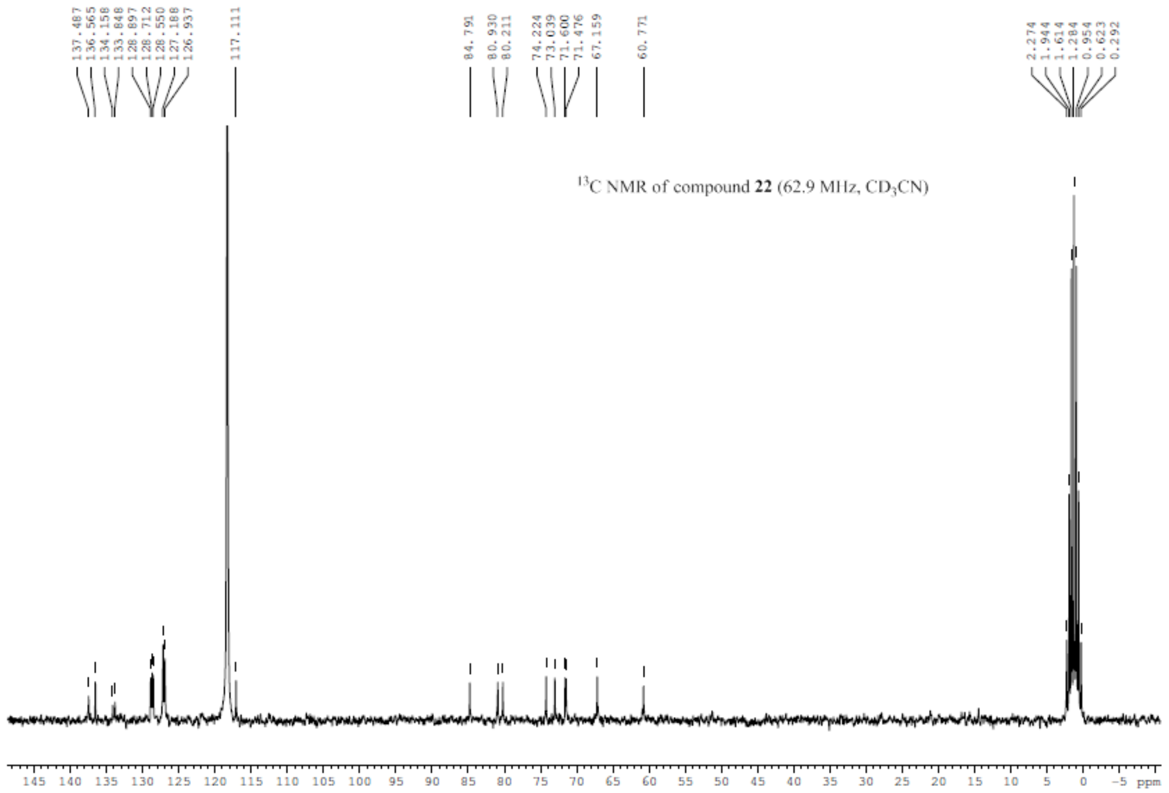

137.487
136.565
134.158
133.848
128.897
128.712
128.550
127.188
126.937
117.111
84.791
80.930
80.211
74.224
73.039
71.600
71.476
67.159
60.771
2.274
1.944
1.614
1.284
0.954
0.623
0.292
^{13}C NMR of compound **22** (62.9 MHz, CD_3CN)
145 140 135 130 125 120 115 110 105 100 95 90 85 80 75 70 65 60 55 50 45 40 35 30 25 20 15 10 5 0 -5 ppm
